# Enteric polymers beyond protection: mechanistic insights and translational implications for supersaturating oral drug delivery systems

**DOI:** 10.1016/j.ijpx.2026.100689

**Published:** 2026-10-17

**Authors:** Vishvesh Joshi, Lokesh Yadav, Yalamanda Varla, Pranav Patel, Yagni Shah, Arvee Prajapati, Vijaykumar Chaudhary, Rikin Patel, Praveen Thaggikuppe Krishnamurthy, Sonam Patel, Derajram Benival

**Affiliations:** a Chartwell Pharmaceuticals LLC, 77 Brenner Dr, Congers, NY, 10920, USA; b Department of Pharmaceutical Chemistry, College of Pharmacy, JSS University, Noida, Uttar Pradesh, 201301, India; c Department of Pharmaceutics, National Institute of Pharmaceutical Education and Research -Ahmedabad (NIPER-A), Palaj, Gandhinagar, Gujarat, 382355, India; d Intas Pharmaceuticals, Ahmedabad, Gujarat, 380054, India; e Department of Pharmacology, College of Pharmacy, JSS University, Noida, Uttar Pradesh, 201301, India

**Keywords:** Amorphous solid dispersions, Enteric polymers, Hypromellose acetate succinate (HPMCAS), Supersaturation, Precipitation inhibition, Drug-rich colloids, Oral bioavailability

## Abstract

Poor aqueous solubility continues to limit oral delivery of many drugs. Weak bases are particularly challenging because they may dissolve in the stomach and subsequently precipitate as the gastrointestinal pH increases. Enteric polymers are therefore being explored not only as protective coatings but also as functional carriers in amorphous solid dispersions (ASDs).

This review looks at enteric polymers from the perspective of spatiotemporal regulation of supersaturation, encompassing where drug release begins and what happens to the drug after release. The discussion covers HPMCAS, HPMCP, and methacrylate-based polymers including Eudragit L100-55, Eudragit L100, and Kollicoat MAE 100-55. Published studies on drug release, precipitation, phase behavior, membrane transport, and pharmacokinetics are considered together with regulatory information for selected marketed products.

Enteric polymers do not produce uniform effects across formulations. Depending on the drug, polymer grade, drug loading, dissolution conditions, and manufacturing process, they may modulate gastric drug release, influence pH-shift precipitation, and affect nucleation, crystal growth, or drug-rich colloidal phases. However, increased apparent drug concentration does not necessarily indicate a greater concentration of molecularly dissolved drug, membrane transport, or systemic exposure.

The review also considers manufacturing-related factors, including spray drying and hot-melt extrusion, and outlines a mechanism-based framework for selecting enteric polymers according to drug-specific biopharmaceutical risks, drug–polymer compatibility, and practical development requirements. Overall, enteric polymers should be selected based on formulation-specific evidence rather than the assumption that pH-dependent release will inherently improve oral bioavailability.

## Graphical abstract

**Figure:**
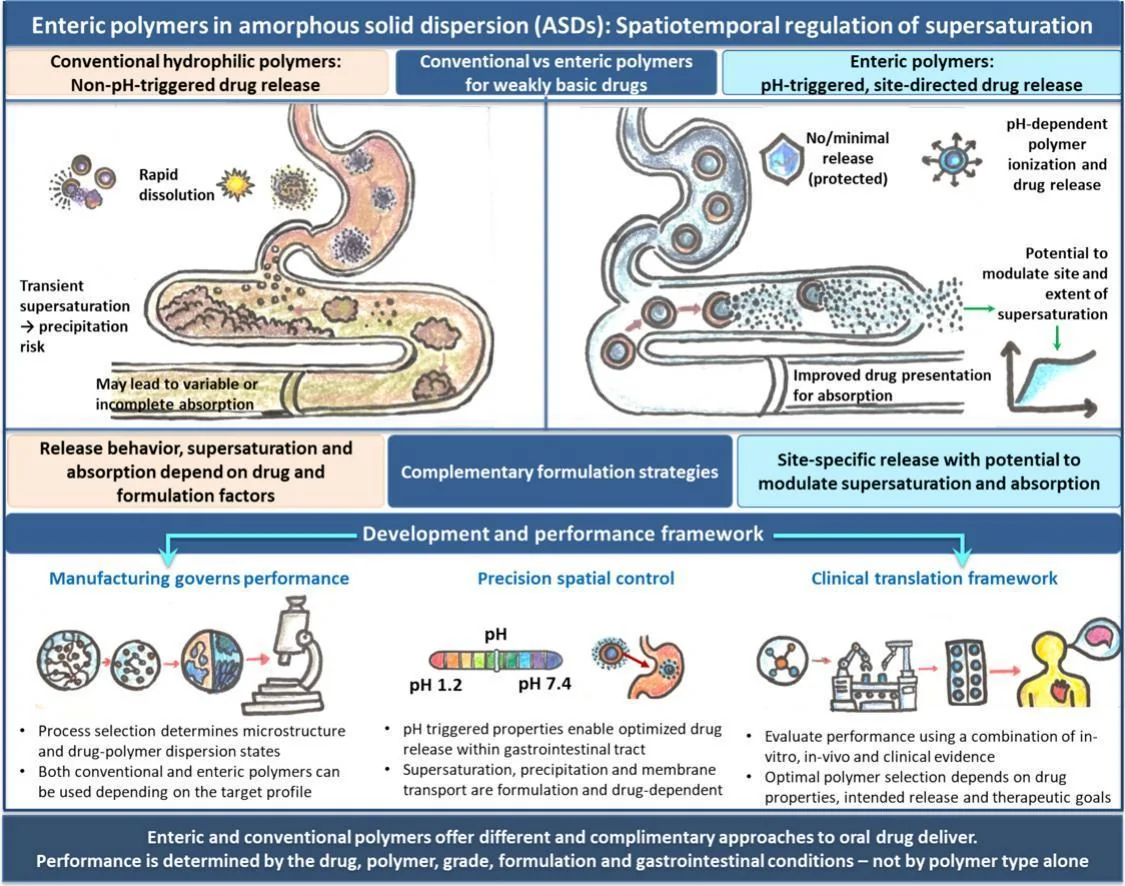

## 1 Introduction

Oral administration remains the preferred route for many small-molecule drugs because of patient convenience, ease of manufacturing, and commercial scalability (Homayun et al., 2019). However, a large number of such drugs exhibit poor aqueous solubility and dissolution rate limited absorption (Pandi et al., 2020). The long-recognized trend toward higher lipophilicity in medicinal chemistry, originally articulated through the “Rule of Five” framework, continues to influence modern drug discovery pipelines. As a result, a substantial proportion of drug compounds fall under Biopharmaceutics Classification System (BCS) Class II or IV (Kumari et al., 2023; Lipinski, 2000). For these drugs, systemic exposure is frequently governed by the ability to generate and maintain adequate concentrations within the gastrointestinal tract (Brouwers et al., 2009; Gan et al., 2023).

While the BCS framework provides regulatory guidance for bioequivalence (Amidon et al., 1995; Waiver of In Vivo Bioavailability and Bioequivalence Studies for Immediate-Release Solid Oral Dosage Forms Based on a Biopharmaceutics Classification System - Digital Collections - National Library of Medicine, 2017), it offers limited direction for formulation strategy in the case of drugs exhibiting poor dissolution rate (drugs belonging to Developability Classification System; DCS class IIa) and low solubility (DCS class IIb). In such cases, formulation scientists must rely on enabling technologies to address these limitations. Among these, formulating amorphous solid dispersions (ASDs) and related supersaturating systems have emerged as effective strategies for enhancing oral bioavailability of poorly aqueous soluble drugs (Baghel et al., 2016; Pandi et al., 2020; Tambe et al., 2022).

Amorphization increases the free energy of the drug substance, thereby enhancing apparent solubility and dissolution rate relative to its crystalline solid form (Alonzo et al., 2010; Hancock and Zografi, 1997). However, the resulting supersaturated solution is thermodynamically unstable and highly susceptible to precipitation. The generation and maintenance of supersaturation in an aqueous environment by amorphous systems is commonly described by the ‘spring and parachute’ effect, wherein rapid drug dissolution (‘spring’) produces a transient supersaturated state, followed by finite precipitation inhibition (‘parachute’) which maintains elevated drug concentrations (Augustijns and Brewster, 2012; Brouwers et al., 2009; Taylor and Zhang, 2016). In ASDs, polymers can influence molecular mobility, inhibit nucleation, and retard crystal growth, thereby supporting sustained supersaturation under appropriate conditions and potentially enhancing drug absorption through passive diffusion (Alonzo et al., 2011; Qian et al., 2010).

Conventional pH-independent hydrophilic polymers such as polyvinylpyrrolidone (PVP), copovidone (PVP-VA), high molecular weight polyethylene glycol (PEG), and hydroxypropyl methylcellulose (HPMC) have been widely employed as carriers in ASDs to improve dissolution rate and maintain supersaturation in aqueous media of poorly water soluble drugs (Bhujbal et al., 2021; Paudel et al., 2013). These hydrophilic polymers dissolve readily in gastric fluid and generate supersaturated drug solutions. However, this supersaturated solution, upon transition from stomach into the intestine, is susceptible to recrystallization or precipitation particularly for weakly basic drugs (Gan et al., 2023; Kostewicz et al., 2004). During gastric emptying and subsequent pH shift, drug precipitation may occur in the intestinal environment, potentially limiting the availability of the soluble fraction of the drug at the major absorption site i.e., small intestine (Gan et al., 2023; Kostewicz et al., 2014).

Enteric polymers were originally developed as pH-dependent coating materials that prevent drug release in the gastric fluid and facilitate dissolution at higher intestinal pH (Maderuelo et al., 2019). Historically, these polymers have been used primarily to protect drugs from gastric acid-induced degradation, although their potential contributions in controlling drug release and absorption have been recently recognized (Maderuelo et al., 2019). Increasingly, enteric polymers are also being incorporated as matrix-forming carriers within ASDs rather than solely as external coating materials. This transition reflects a growing recognition that their pH-dependent solubility, drug–polymer interactions, and precipitation inhibition properties may collectively influence the location and persistence of supersaturation within the gastrointestinal tract. However, the mechanistic basis and translational significance of these functions remain incompletely synthesized. When incorporated directly into ASDs, these polymers exhibit physicochemical properties which extend beyond gastric protection. These include pH-triggered dissolution, reduced hygroscopicity and enhanced physical stability in comparison to commonly used hydrophilic polymers, and the ability to form drug–polymer interactions that can contribute to supersaturation stabilization under intestinal conditions (Friesen et al., 2008; Ilevbare et al., 2012; Pandi et al., 2020; Tanno et al., 2004).

Previous reviews have addressed individual aspects of this field, including HPMCAS as an ASD carrier, ASD manufacturing approaches, supersaturation and precipitation behavior, and marketed ASD products. (Butreddy, 2022; Corrie et al., 2023; Moseson et al., 2024). The distinctive contribution of the present review is to integrate these elements within a single mechanistic and translational framework focused on the spatiotemporal regulation of supersaturation. Rather than treating delayed release or prolonged supersaturation as surrogate measures of improved oral performance, the framework considers a sequence of linked but distinct events: where and when drug release occurs; whether release generates and maintains supersaturation or drug-rich phases; how much drug remains molecularly dissolved and available for membrane transport; and whether these effects are ultimately reflected in systemic exposure. The review therefore connects polymer physicochemical properties and gastrointestinal release behavior with phase behavior, membrane transport, pharmacokinetics, manufacturing, and formulation selection. This synthesis is further translated into a mechanism-based decision framework that identifies when an enteric-polymer ASD may provide a meaningful advantage and when a conventional ASD or another formulation strategy may be more appropriate.

### 1.1 Literature identification and evidence interpretation

This article was prepared as a structured narrative review. PubMed and Google Scholar were used as the primary bibliographic databases, with the main search period covering January 2000 to August 2026. Additional publications were identified from reference lists, articles citing key studies, and targeted searches on journal or publisher websites. Search terms were combined in different ways depending on the topic and included ASD, enteric polymer, HPMCAS, hydroxypropyl methylcellulose phthalate (HPMCP), cellulose acetate phthalate, methacrylic acid copolymer, Eudragit, Kollicoat MAE, supersaturation, precipitation inhibition, pH shift, liquid–liquid phase separation, drug-rich colloids, membrane flux, bioavailability, spray drying, and hot-melt extrusion. For marketed products, information was checked against FDA, DailyMed, and EMA documents whenever suitable primary sources were available.

Because this was a narrative review, formal systematic-review inclusion and exclusion criteria were not applied. Instead, studies were selected according to their relevance to the questions addressed in the review. Greater attention was given to studies that directly compared polymers or polymer grades, examined behavior after a gastric-to-intestinal pH change, or measured precipitation, phase separation, membrane transport, or pharmacokinetic performance. Studies dealing mainly with conventional enteric coating were used where they helped explain polymer chemistry or pH-dependent dissolution, but they were not taken as evidence that the same polymer would improve absorption when used as an ASD carrier. Regulatory documents were used mainly to confirm product composition, polymer use, manufacturing information when publicly available, and other product-specific details.

The published studies also differ considerably in drug loading, polymer level, dissolution medium, pH-shift conditions, manufacturing method, and analytical technique. For this reason, numerical results from different studies were not combined into a quantitative meta-analysis. Instead, the evidence was interpreted according to what was actually measured. Dissolution, supersaturation, precipitation, and phase-behavior studies were treated mainly as mechanistic in vitro evidence. Permeability or membrane-flux experiments were used to assess whether the available drug could contribute to transport across a membrane. Animal pharmacokinetic studies were treated as preclinical evidence that formulation effects were reflected in systemic exposure, while human pharmacokinetic studies were given greater weight when considering clinical relevance. Regulatory and marketed-product information was considered separately because approval shows that a formulation approach can be successfully developed and used clinically, but it does not by itself prove that a particular polymer mechanism is responsible for the observed clinical performance.

## 2 Mechanistic basis: enteric polymers as spatiotemporal modulators of supersaturation

### 2.1 Spatial control of drug release through pH-triggered dissolution

The defining functional characteristic of enteric polymers is their pH-dependent solubility profile. Unlike hydrophilic polymers such as high molecular weight PEG, PVP, copovidone, or HPMC, which dissolve readily under gastric conditions, enteric polymers remain largely unionized and insoluble at acidic pH and undergo dissolution only upon ionization at intestinal pH. This ionization associated solubility enables spatial control over matrix dissolution and drug release, thereby delaying supersaturation generation until the particles of ASD reach into the small intestine (Nguyen et al., 2023b; Zhang et al., 2022). The location of supersaturation generation is further influenced by the dissolution threshold of the enteric polymer. For example, different HPMCAS grades exhibit distinct dissolution pH ranges based on cited nominal threshold of approximately pH 5.5, 6.0 and ≥ 6.5 for grades L, M and H respectively, allowing formulation scientists to modulate the onset of drug release within different regions of the small intestine. Consequently, polymer selection should consider not only drug properties but also the desired site of supersaturation generation relative to the drug absorption window.

For poorly soluble weak bases, this spatial release control has important mechanistic implications. Weakly basic compounds exhibit pH-dependent solubility governed by the Henderson–Hasselbalch relationship, resulting in high apparent solubility in the acidic pH (stomach) due to protonation, followed by a substantial reduction in solubility and/or precipitation upon transition into the small intestine (existence of near neutral pH) (Gan et al., 2023; Kostewicz et al., 2004). In conventional hydrophilic ASD systems, early gastric release may therefore generate transient supersaturation that may subsequently lead drug to precipitate during intestinal pH shift, as the solubility threshold decreases, thereby reducing the soluble fraction available for absorption, as illustrated in Fig. 1 (Brouwers et al., 2009; Gan et al., 2023). Although this mechanism is most relevant for weakly basic drugs because of their pronounced pH-dependent solubility, similar advantages may also be observed for selected neutral compounds exhibiting rapid crystallization or precipitation following dissolution. However, the magnitude of benefit remains highly drug dependent and should be evaluated on a case-by-case basis.

**Fig. 1:**
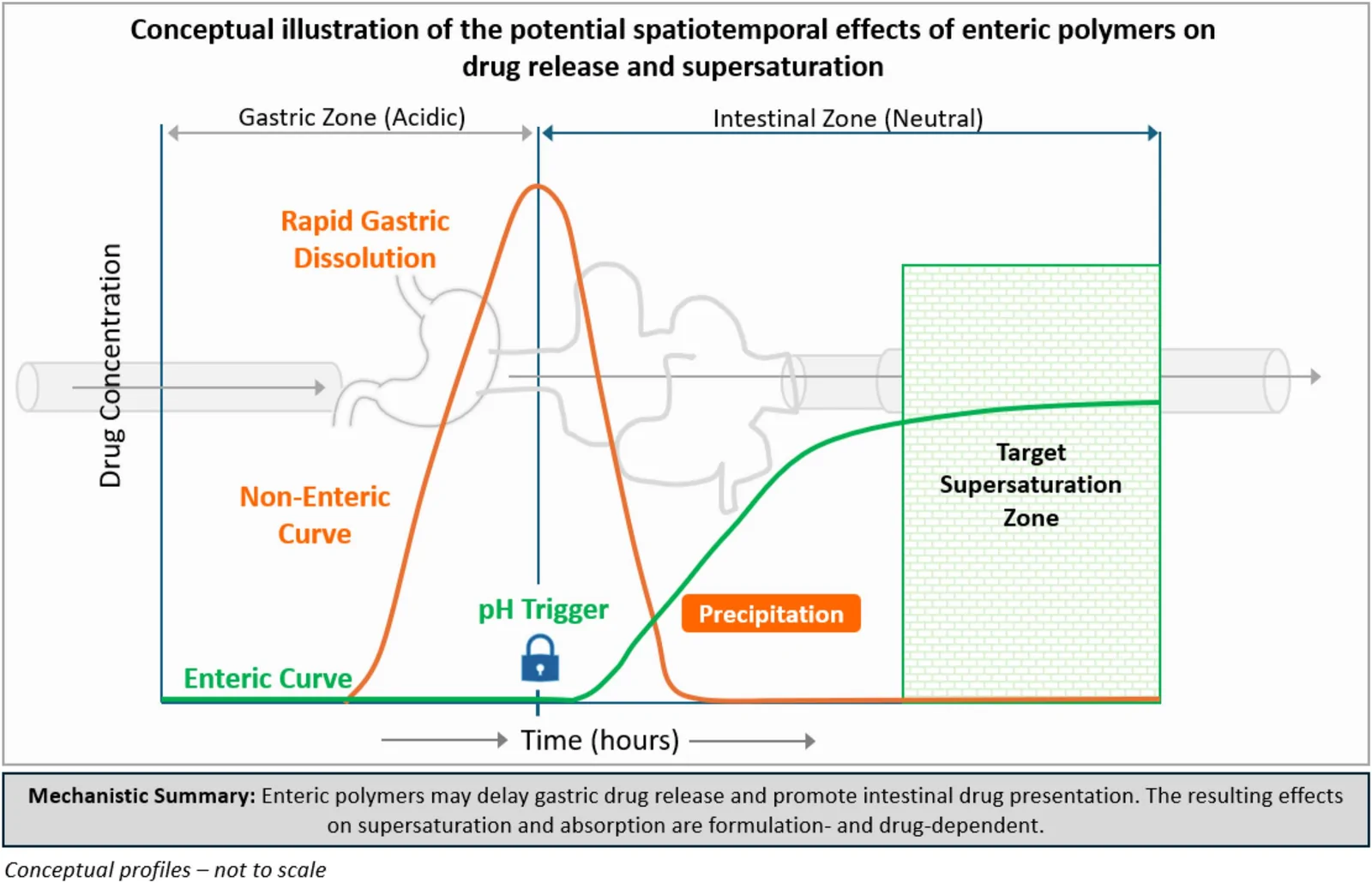
Illustration of the mechanistic distinction between conventional hydrophilic ASD systems and enteric polymer-based ASDs with respect to the location of supersaturation generation and precipitation following gastric-to-intestinal pH transition. Original conceptual illustration prepared by the authors specifically for this review; not reproduced or adapted from previously published material.

Consequently, a temporary mismatch can arise between the generation of supersaturation and the site of optimal drug absorption. Enteric polymer-based ASDs, by delaying drug release in the stomach, may reduce this mismatch and thereby possibly improve the temporal alignment between supersaturation and drug absorption (Kostewicz et al., 2014). In addition to the decrease in solubility due to precipitation, salts of weak bases may undergo ‘salt-to-free base’ conversion during intestinal transit, often resulting in rapid crystallization and loss of supersaturation (Nguyen et al., 2023b). This conversion is governed by the pH-dependent solubility behavior of the weak bases encountered in the gastrointestinal tract (often described by pH_max_), where the free base which has lower solubility in comparison to salt form/ionized form predominates at the intestinal pH (Nguyen et al., 2023b).

Further, emerging evidence suggests that non-classical pathways such as liquid–liquid phase separation (LLPS) may precede crystallization. In this process, nanodroplets of drugs are formed when concentrations exceed the amorphous solubility threshold. These nanodroplets may act as transient reservoirs that replenish the molecularly dissolved drug fraction during intestinal absorption (Kawakami, 2025). In the case of enteric polymer-based ASDs, minimizing polymer dissolution and drug leaching at low gastric pH (e.g., with HPMCAS) may mitigate premature supersaturation and reduce the likelihood of acid-induced phase transformations (Nguyen et al., 2023a, Nguyen et al., 2023b). Several HPMCAS-based ASD studies have demonstrated prolonged supersaturation in biorelevant intestinal media (Kraft et al., 2014). For example, the HPMCAS-based posaconazole delayed-release tablet (Noxafil®) was specifically designed to suppress drug release in the stomach and promote dissolution following intestinal pH transition (refer to the detailed explanation in Section 4). Likewise, mechanistic studies on delamanid ASDs prepared with enteric polymers (HPMCP or HPMCAS) showed that premature exposure to acidic media compromised subsequent intestinal drug release, leading to precipitation, whereas limiting gastric release preserved the supersaturation potential after transfer to intestinal conditions leading to availability of free drug for subsequent absorption. These examples illustrate how enteric polymers may function not only as precipitation inhibitors but also as regulators of the spatial and temporal generation of supersaturation within the gastrointestinal tract (Budiman et al., 2025; Nguyen et al., 2023a).

Although pH-triggered release does not universally guarantee improved bioavailability, physiological variables including food intake, gastric emptying, intestinal pH, and acid-reducing therapy may alter polymer dissolution and the timing of supersaturation generation. Consequently, these factors should be considered during formulation development and evaluated using biorelevant dissolution methodologies (Augustijns and Brewster, 2012; Gan et al., 2023; Kostewicz et al., 2014). While pH-triggered dissolution determines where supersaturation is generated within the gastrointestinal tract, sustained oral absorption ultimately depends on the ability of the polymer to maintain that supersaturated state following intestinal release.

### 2.2 Supersaturation stabilization mechanisms

While spatial control of drug release represents a key functional distinction of enteric polymers, their contribution extends beyond pH-triggered dissolution. Following ionization and dissolution under intestinal conditions, these polymers may contribute to the maintenance of supersaturation through complementary molecular, interfacial, thermodynamic, kinetic, diffusional, and solid-state mechanisms. These contributions include molecular drug–polymer interactions, interfacial stabilization, modulation of drug activity, suppression of nucleation and crystal growth, diffusion and viscosity effects, and stabilization of the amorphous solid-state (Alqahtani et al., 2021; Qian et al., 2021; Taylor and Zhang, 2016). Because supersaturation is intrinsically metastable, its persistence depends on the balance among drug activity, molecular mobility, nucleation kinetics, and crystal-growth rates (Alonzo et al., 2011; Gan et al., 2023; Ueda et al., 2025).

Common enteric polymers, including HPMCAS, HPMCP, cellulose acetate phthalate, and methacrylic acid copolymers, contain ionizable carboxylic acid groups that undergo progressive deprotonation as the environmental pH increases toward intestinal conditions (Friesen et al., 2008; Zhang et al., 2022). Ionization enhances polymer solubility and introduces electrostatic repulsion, while also modifying intermolecular interactions with dissolved drug molecules (Friesen et al., 2008; Ueda et al., 2019). These effects are not identical across all enteric polymers. Differences in substitution chemistry, molecular weight, ionization behavior, hydrophobicity, and polymer grade influence dissolution, drug–polymer compatibility, interfacial activity, and precipitation-inhibiting performance. Supersaturation stabilization should therefore be regarded as a polymer- and grade-dependent property rather than a generic characteristic of enteric polymers.

Molecular drug–polymer interactions represent an important stabilization mechanism. Hydrogen bonding, hydrophobic association, and, where applicable, electrostatic interactions can reduce drug activity and molecular mobility and interfere with the molecular organization required for crystallization (Ilevbare et al., 2012). In cellulose-derived enteric polymers such as HPMCAS, HPMCP, and cellulose acetate phthalate, succinoyl or phthalyl substituents provide ionizable and hydrogen-bonding functionalities that can associate with complementary drug moieties. Methacrylic acid copolymers can provide similar ionization and hydrogen-bonding capacity through carboxylic acid groups present within a different polymer backbone. These interactions may occur in the solid dispersion, in solution, and at drug–polymer interfaces, creating conditions less favorable for phase separation and crystallization.

The likelihood and strength of drug–polymer interactions may be assessed during preformulation using complementary computational and experimental approaches, including Hansen solubility parameters, Flory–Huggins interaction parameters, COSMO-RS calculations, Fourier-transform infrared spectroscopy, and solid-state nuclear magnetic resonance spectroscopy. These methods can support rational polymer selection, although experimental confirmation under relevant dissolution conditions remains necessary.

Enteric polymers may also exhibit amphiphilic behavior because of the coexistence of hydrophobic substituents and hydrophilic or ionizable groups (Elkhabaz et al., 2019; Friesen et al., 2008). This amphiphilic character may promote preferential adsorption at the interface of emerging drug-rich domains or crystal nuclei. Interfacial adsorption may lower interfacial energy, reduce droplet coalescence, block active crystal-growth sites, and hinder further molecular organization (Elkhabaz et al., 2019; Ilevbare et al., 2012).

From a thermodynamic perspective, polymer association with dissolved drug may reduce drug activity or alter its apparent chemical potential, thereby decreasing the driving force for crystallization (Ilevbare et al., 2012; Shah and Taylor, 2025; Taylor and Zhang, 2016). From a kinetic perspective, polymer adsorption onto emerging nuclei or growing crystal surfaces may increase the nucleation barrier and retard crystal growth (Bapat et al., 2024; Elkhabaz et al., 2019; Ilevbare et al., 2012). The relative contribution of these mechanisms varies among systems. In some formulations, specific molecular interactions may dominate, whereas in others, interfacial adsorption, diffusion limitation, or phase-behavior modulation may be more important (Alonzo et al., 2011; Gan et al., 2023; Qian et al., 2021). Polymer molecular weight can further influence supersaturation stabilization through chain dimensions, interfacial coverage, solution viscosity, and diffusion restriction. Higher-molecular-weight polymers may exhibit greater chain entanglement and produce higher local viscosity, thereby slowing molecular rearrangement and transport of drug molecules toward growing crystal surfaces. However, the relationship is not necessarily monotonic. At an equivalent mass concentration, increasing molecular weight reduces the number of individual polymer chains available in solution and may also slow polymer dissolution. Consequently, stabilization may depend on an optimal balance among chain length, polymer concentration, dissolution rate, interfacial adsorption, and specific drug–polymer interactions rather than on selection of the highest-molecular-weight grade (Alonzo et al., 2011; Indulkar et al., 2016; Odeh et al., 2023; Vora et al., 2016).

Substitution pattern is particularly important for cellulose-derived enteric polymers because it governs the balance among ionization, hydrophobicity, hydrogen-bonding capacity, dissolution-pH threshold, and interfacial activity. In HPMCAS, the relative proportions of hydrophobic acetyl and ionizable succinoyl groups influence polymer hydration, dissolution, drug association, and adsorption at drug-rich interfaces. Greater ionizable substitution may facilitate hydration and dissolution once the threshold pH is reached, whereas greater hydrophobic substitution may strengthen association with poorly soluble drugs or drug-rich phases but can also delay polymer dissolution. Similarly, the degree and distribution of phthalyl substitution in HPMCP and cellulose acetate phthalate influence ionization and hydrophobic association. Accordingly, substitution pattern affects both where supersaturation is generated and how effectively it is stabilized (Friesen et al., 2008; Mittal et al., 2025; Ueda et al., 2013, Ueda et al., 2019).

Experimentally, the influence of enteric polymers on crystallization kinetics can be evaluated by measuring nucleation induction time, precipitation onset, crystal-growth rate, supersaturation duration, and drug concentration–time profiles (Alonzo et al., 2010; Yamashita and Sun, 2019). These parameters may be assessed using pH-shift or transfer studies, turbidity or light-scattering measurements, ultraviolet or Raman spectroscopy, microscopy, particle-size analysis, and solid-state techniques such as powder X-ray diffraction (Alonzo et al., 2010; Francine et al., 2012; Moseson et al., 2021). Because each method probes a different aspect of the crystallization process, complementary techniques are generally required to distinguish delayed nucleation from reduced crystal-growth rate (Alonzo et al., 2010; Moseson et al., 2021).

Solid-state properties of the ASD before dissolution also influence subsequent supersaturation behavior. Enteric polymers with suitable glass-transition temperatures (Tg) may restrict molecular mobility within the amorphous matrix and reduce the risk of phase separation or recrystallization during storage (Alonzo et al., 2010; Ueda et al., 2013). Drug–polymer miscibility, drug loading, residual solvent, moisture content, and processing history determine the initial microstructure of the dispersion. The initial homogeneity of the ASD therefore influences subsequent dissolution, phase separation, and supersaturation behavior. Appropriate polymer grade and substitution pattern may provide a balance among drug miscibility, physical stability, controlled intestinal dissolution, and interfacial stabilization (Friesen et al., 2008; Ueda et al., 2013, Ueda et al., 2019).

The stabilization capacity of enteric polymers is strongly drug-dependent. Current evidence is most compelling for weakly basic compounds with pronounced pH-dependent solubility and a high tendency to precipitate following gastric-to-intestinal transfer. Application of these concepts to neutral compounds or weak acids should be evaluated using drug-specific properties, including ionization behavior, lipophilicity, hydrogen-bonding functionality, lattice strength, and crystallization tendency (Alonzo et al., 2011; Gan et al., 2023; Ilevbare et al., 2012; Indulkar et al., 2016; Qian et al., 2021; Ueda et al., 2019). Consequently, not all poorly water-soluble drugs derive the same degree of benefit from enteric polymer-based ASDs.

Overall, supersaturation stabilization should be regarded as an emergent property of the drug–polymer–formulation system rather than an inherent property of the polymer alone. However, maintenance of total or apparent drug concentrations, including concentrations associated with drug-rich phases or colloidal structures, does not necessarily indicate an increase in the molecularly dissolved fraction, membrane flux, or systemic exposure. These relationships therefore require evaluation using complementary dissolution, phase-behavior, permeation, and, where appropriate, pharmacokinetic studies.

### 2.3 Drug-rich phase formation and colloidal stabilization

When supersaturation exceeds a critical threshold, the system may undergo LLPS, which may lead to the formation of drug-rich nanodroplets with drug concentrations approaching amorphous solubility (Qian et al., 2021; Ueda et al., 2019, Ueda et al., 2025). These transient nanodroplets may act as reservoirs that replenish the molecularly dissolved fraction, thereby prolonging apparent supersaturation. However, the extent to which this process contributes to intestinal absorption depends on the rate of drug exchange between colloidal and molecularly dissolved states. Conversely, drug retained within a relatively stable or poorly permeable colloidal phase may not be immediately available for membrane transport. Therefore, the formation or persistence of drug-rich phases should not be interpreted as direct evidence of improved intestinal absorption (Bapat et al., 2024; Elkhabaz et al., 2019; Indulkar et al., 2016; Qian et al., 2021; Ueda et al., 2025).

Cryogenic transmission electron microscopy (cryo-TEM), dynamic light scattering (DLS), and small-angle scattering techniques have provided structural evidence for the formation, morphology, and persistence of the drug-rich colloidal phases in HPMCAS-based ASD systems (Nunes et al., 2022; Ricarte et al., 2017). Together, these findings suggest that enteric polymers may influence both crystallization and persistence of transient colloidal structures during dissolution (Shah and Taylor, 2025; Ueda et al., 2013, Ueda et al., 2014).

From an absorption perspective, the existence of drug-rich colloids requires careful interpretation of measured drug concentrations. Total or apparent drug concentration may include both molecularly dissolved drug and drug associated with colloids or drug-rich droplets. In contrast, the molecularly dissolved fraction is more directly relevant to passive membrane permeation. Therefore, an increase in apparent supersaturation does not necessarily result in a proportional increase in membrane flux or systemic exposure (Elkhabaz et al., 2019; Stewart et al., 2017). While passive diffusion across the intestinal epithelium is primarily driven by the free molecular fraction, drug-rich phases may act as a buffering reservoir, continuously replenishing dissolved drug concentration as permeation proceeds (Qian et al., 2021; Stewart et al., 2017). Whether drug-rich nanodroplets directly contribute to epithelial permeation or primarily function by replenishing the molecularly dissolved fraction remains an area of active investigation.

Evidence from experimental systems illustrates that these effects may vary according to drug, polymer, and test conditions. Stewart et al. reported that membrane-flux rankings for itraconazole/HPMCAS spray-dried dispersions were associated with the maximum absorption rate observed in rats, suggesting that drug-rich colloids may contribute to drug transport under the conditions tested (Stewart et al., 2017). However, other studies have reported that higher apparent supersaturation does not necessarily result in greater membrane transport. Elkhabaz et al. observed that supersaturation measured in biorelevant or aspirated intestinal fluids was not consistently associated with increased membrane transport, partly because a proportion of the measured drug was associated with colloidal structures rather than being available as molecularly dissolved drug (Elkhabaz et al., 2019). Studies evaluating different HPMCAS grades have further indicated that polymer–drug association, colloidal-phase characteristics, and drug concentration can influence membrane transport in a context-dependent manner (Ueda et al., 2025; Ueda and Taylor, 2020).

Importantly, the formation and stabilization of drug-rich phases are functions of both drug and polymer properties. Factors such as polymer chemistry, substitution pattern, ionization behavior, and their effects on amorphous solubility and colloidal droplet properties may determine whether stable drug-rich phases form and how long they persist (Ueda et al., 2014, Ueda et al., 2025; Ueda and Taylor, 2020). Compared with pH-independent water soluble hydrophilic polymers, enteric polymers such as HPMCAS may provide different levels of droplet stabilization under intestinal pH conditions due to its amphiphilic, weakly acidic structure which can modulate amorphous solubility, colloidal phase behavior, and crystallization inhibition (Shah and Taylor, 2025; Ueda et al., 2014; Ueda and Taylor, 2020). Thus, optimal performance requires an appropriate balance between droplet stability and efficient drug exchange between colloidal and molecular states (Elkhabaz et al., 2019; Stewart et al., 2017). These observations indicate that enteric polymers may influence not only precipitation inhibition but also the dynamic evolution of drug-rich colloidal phases, thereby linking dissolution behavior with availability of free drug for absorption (Pigliacelli et al., 2023; Qian et al., 2021; Stewart et al., 2017; Ueda et al., 2014, Ueda et al., 2019).

### 2.4 Moisture interactions and physical stability

While dissolution rate and amorphous solubility enhancement are central to the performance of ASDs, their long-term performance is equally dependent on solid-state stability during storage and processing. Moisture is one of the most significant destabilizing factors for ASDs because water acts as a plasticizer, lowering the Tg, increasing molecular mobility, and consequently promoting phase separation or recrystallization. Accordingly, the interaction between enteric polymers and water becomes a critical determinant of whether an ASD remains stable during shelf life (Chen et al., 2018; Lehmkemper et al., 2017; Patel et al., 2022; Patel and Serajuddin, 2022).

Hygroscopic carriers, specifically water-soluble, pH-independent hydrophilic polymers, may therefore exhibit pronounced Tg reductions under elevated relative humidity, and thereby increase crystallization risk during storage (Lehmkemper et al., 2017; Patel et al., 2022; Patel and Serajuddin, 2022). On the contrary, enteric polymers often display distinct water sorption behavior due to their substitution chemistry and amphiphilic characteristics (Friesen et al., 2008; Patel et al., 2022; Ueda et al., 2014). This moisture sorption attribute can influence how rapidly Tg is depressed and, consequently, how quickly molecular mobility increases under humid environment. In practice, it conveys that, depending on formulation composition and storage conditions, some enteric polymer matrices may better preserve kinetically stable glassy states and delay moisture triggered phase separation (Friesen et al., 2008; Lehmkemper et al., 2017; Patel et al., 2022).

From a translational perspective, controlling moisture exposure is not solely a packaging concern but a broader formulation and process design consideration. Polymer type and grade selection, drug loading optimization, manufacturing method selection and control of the physical state during processing and storage are all necessary to balance dissolution performance with long-term stability (Butreddy, 2022; Lehmkemper et al., 2017; Sarabu et al., 2020). In this context, enteric polymers offer a potentially useful combination of pH-triggered release and differentiated polymer-dependent moisture–Tg behavior.

The mechanistic differences between conventional pH-independent hydrophilic water soluble polymers and enteric polymer-based ASDs are illustrated in Fig. 2.

**Fig. 2:**
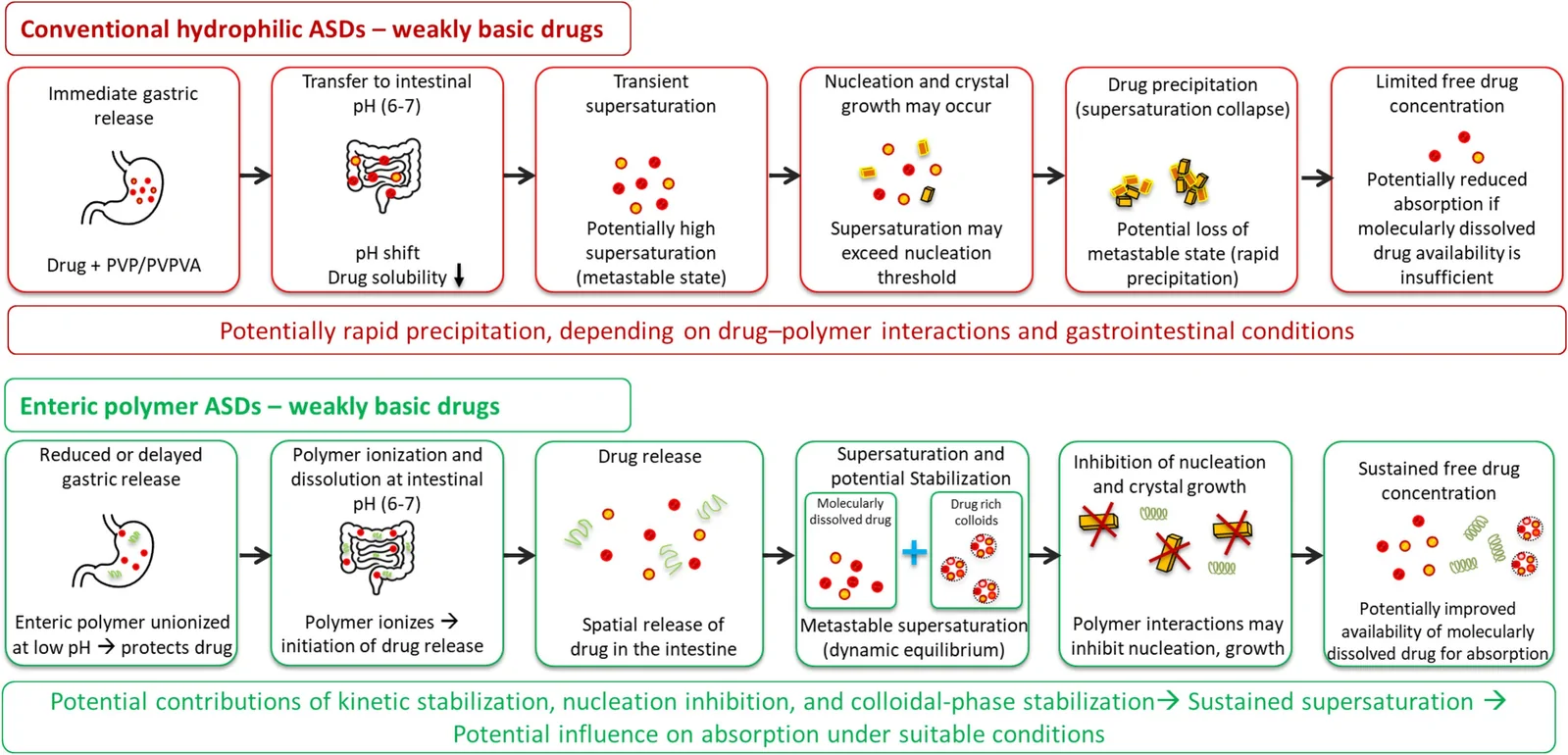
Conceptual comparison of the potential effects of conventional hydrophilic and enteric-polymer ASDs for weakly basic drugs. Conventional water-soluble polymers may permit drug release before or during the gastric-to-intestinal pH transition, after which supersaturation, precipitation, and molecularly dissolved drug availability depend on drug–polymer interactions and gastrointestinal conditions. Enteric polymers may reduce or delay gastric drug release and shift drug presentation toward the intestinal environment, where polymer ionization, drug–polymer interactions, polymer aggregation, and drug-rich-phase behavior may influence dissolution and supersaturation. Drug-rich colloidal phases may contribute to drug transport in certain systems, but apparent supersaturation or colloidal persistence does not necessarily predict membrane flux or systemic exposure. These effects are drug-, polymer-, and formulation-specific (Bristol et al., 2021; Curatolo et al., 2009; Li and Taylor, 2018; Stewart et al., 2017; Ueda and Taylor, 2020). Original conceptual illustration prepared by the authors specifically for this review; not reproduced or adapted from previously published material.

## 3 Enteric versus non-enteric polymers in ASDs

The primary distinction is that conventional hydrophilic carriers generally permit polymer and drug release over a broad gastrointestinal pH range, whereas enteric polymers introduce a pH-dependent release threshold. Consequently, conventional polymers are often suited to formulations requiring rapid supersaturation generation, while enteric polymers may be advantageous when drug release should be delayed until intestinal conditions are reached. This difference provides a potential means of modulating the site and timing of supersaturation rather than simply maximizing its initial magnitude. The latter consideration is particularly relevant for weakly basic drugs susceptible to precipitation during gastric-to-intestinal transfer, although the potential functions of enteric polymers are not restricted to this drug class. Their performance in neutral, weakly acidic, or weakly ionizable compounds may instead arise from differences in matrix dissolution, precipitation inhibition, dissolution phase behavior, or solid-state stabilization. These effects are drug-, polymer-, grade-, and medium-dependent and should not be interpreted as evidence of universal superiority over conventional hydrophilic polymers. The main functional distinctions between enteric and non-enteric polymer systems are summarized in Table 1.

**Table 1:** Representative enteric polymers relevant to ASD design and spatiotemporal supersaturation control.

Polymer / representative grade	Backbone and ionizable group	Nominal dissolution behavior^a^	Potential ASD implications	Strength of ASD evidence / practical note
HPMCAS L / M / H	Cellulose ether ester; succinoyl carboxyl groups plus acetyl substitution	Approx. ≥ 5.5 / ≥ 6.0 / ≥ 6.5, respectively	Grade tunes release location; substitution also alters hydrophobicity, drug-rich-phase partitioning, amorphous solubility, and crystallization inhibition	Extensive mechanistic and commercial evidence; major enteric ASD platform (Bapat et al., 2024; Butreddy, 2022; Corrie et al., 2023; Friesen et al., 2008; Moseson et al., 2024; Nguyen et al., 2023b; Shah and Taylor, 2025; Ueda et al., 2014; Ueda and Taylor, 2020).
HPMCP HP-50 / HP-55	Hypromellose phthalate; phthalyl carboxyl groups	Approx. ≥ 5.0 / ≥ 5.5	Earlier intestinal ionization than higher-threshold HPMCAS grades; can provide gastric suppression and solution-phase interactions	Moderate ASD evidence; approved HPMCP polymer-matrix product (DailyMed - TOLSURA- Itraconazole Capsule, Gelatin Coated, 2025; TOLSURA® (Itraconazole Capsules), for Oral Use Initial U.S. Approval: 1992, 2024; Monschke and Wagner, 2019; Nguyen et al., 2023b).
Cellulose acetate phthalate (CAP)	Cellulose acetate phthalate; free phthalate carboxyl groups	Typically around pH 6; medium dependent	Enteric protection and pH-triggered ionization; ASD evidence less extensive than HPMCAS	Well established as enteric coating; limited modern head-to-head ASD evidence (Maderuelo et al., 2019).
Eudragit L100-55	Methacrylic acid-ethyl acrylate copolymer (1:1)	Above approx. pH 5.5	Relatively proximal intestinal release; ionic/hydrophobic interactions can stabilize some weak bases; may also interact strongly enough to slow release	Direct ASD evidence with nevirapine, nintedanib, celecoxib systems, ARCC-4, and efonidipine (EUDRAGIT® L *100-55, 2026*; Huang et al., 2026; Monschke and Wagner, 2019; Pöstges et al., 2022, Pöstges et al., 2023; Qin et al., 2022).
Eudragit L100	Methacrylic acid-methyl methacrylate copolymer (1:1)	Above approx. pH 6.0	More distal release than L100-55; strong performance reported for nintedanib but weaker precipitation inhibition in other model systems	Drug-dependent evidence; useful comparator for HPMCAS and L100-55 (EUDRAGIT® L 100, 2026; Ozaki et al., 2013; Qin et al., 2022).
Eudragit S100	Methacrylic acid-methyl methacrylate copolymer with lower acid-group content	Above approx. pH 7.0	Higher threshold may shift release distally and can be unsuitable for drugs with proximal absorption windows	Primarily established for distal intestinal/colonic release; ASD use requires specific justification (EUDRAGIT® S 100, 2026)
Kollicoat MAE 100-55	Methacrylic acid-ethyl acrylate copolymer (1:1)	Above approx. pH 5.5	Chemically suitable for gastric protection and intestinal release; potential ionic/interfacial effects require drug-specific confirmation	Strong coating precedent; comparatively limited peer-reviewed ASD evidence (BASF, 2016).

a Nominal thresholds are formulation and medium dependent and should not be interpreted as sharp in vivo switches.

Acrylate- and methacrylate-based enteric polymers differ from cellulose-based polymers in their polymer backbones and acidic groups, which influence their ionization, dissolution, hydrophobicity, and drug interactions. Eudragit L100-55, Eudragit L100, and Eudragit S100 generally dissolve above approximately pH 5.5, 6.0, and 7.0, respectively. Accordingly, Eudragit S100 is more commonly considered for distal intestinal or colonic release (EUDRAGIT® L 100-55, 2026; EUDRAGIT® L 100, 2026; EUDRAGIT® S 100, 2026). The performance of methacrylate polymers is drug- and formulation-dependent. Although Eudragit L100 or L100-55 maintained supersaturation more effectively than comparator polymers in some studies, Eudragit L100 was less effective than HPMC or PVP in controlling nucleation and crystal growth in other systems (Huang et al., 2026; Ozaki et al., 2013; Qin et al., 2022). Thus, these polymers should be evaluated for individual drugs rather than selected solely on the basis of polymer type. Kollicoat MAE 100-55 also exhibits pH-dependent dissolution and is primarily used as an enteric coating polymer (BASF, 2016). However, compared with HPMCAS and Eudragit L polymers, limited peer-reviewed evidence is available regarding its use in ASDs and supersaturation maintenance. Although it may be considered a potential ASD carrier, current evidence remains limited.

### 3.1 Evidence from direct polymer comparisons

Direct comparisons between enteric and conventional polymers remain relatively limited and are difficult to generalize because performance depends on drug identity, polymer grade and concentration, drug loading, ASD manufacturing method, dissolution medium, and the selected analytical endpoint. The most informative evidence therefore comes from matched studies in which different polymers are evaluated with the same drug under equivalent conditions. Importantly, findings from in vitro dissolution and supersaturation studies should be distinguished from animal pharmacokinetic and human clinical evidence. The representative comparative studies are summarized in Table 2.

**Table 2:** Representative direct-comparison evidence relevant to enteric-polymer ASD selection.

Drug / study	Polymers compared	Experimental context	Main finding	Higher-level evidence	Interpretation
Candesartan cilexetil (Pinto et al., 2018).	HPMC, Soluplus, HPMCAS L/M/H	Supersaturation in two media, including biorelevant conditions	HPMCAS-M strongest overall, but ranking depended on medium	In vitro only	Supports drug- and medium-specific benefit, not class superiority
Danazol (Jackson et al., 2016).	HPMCAS, HPMC, PVP	Nonsink ASD dissolution; LLPS/crystallization and flux characterized across drug loadings	LLPS preceded crystallization in most dispersions; flux indicated a maximum supersaturation near the amorphous-solubility limit, while supersaturation duration changed with polymer and drug loading	In vitro only	Initial concentration alone is an incomplete endpoint
Griseofulvin and danazol (Ozaki et al., 2013).	HPMC, PVP, Eudragit L100	Biorelevant supersaturation testing plus nucleation induction time and seeded crystal-growth measurements	HPMC > PVP > Eudragit L100 for inhibition of nucleation and crystal growth under the tested conditions; effect magnitude differed by drug	In vitro only	Direct counter example to universal enteric superiority
Poorly soluble model compound (Qian et al., 2012).	HPMCAS vs PVPVA	Spray-dried HPMCAS vs PVPVA ASDs; sink/non-sink dissolution, polymer release, residual-matrix crystallinity, and dog PK	Similar apparent in vitro release, but HPMCAS gave better dog exposure; slower HPMCAS dissolution prevented recrystallization within the residual matrix for >24 h	Dog pharmacokinetics	Matrix behavior can matter when conventional dissolution looks similar
Naftopidil (Fukiage et al., 2022).	PVPVA, HPMCAS, Eudragit L100-55	Dissolution/LLPS plus rat oral absorption	Eudragit inhibited LLPS and had poorer conventional dissolution, yet gave greatest rat absorption	Rat pharmacokinetics	More LLPS or higher apparent concentration is not necessarily better
Nintedanib (Qin et al., 2022).	HPMCAS-LG/MG, Eudragit L100-55/L100	pH-shift dissolution (120 min gastric stage followed by pH 6.8 intestinal stage up to 240 min) and rat PK of the selected ASD	Supersaturation ranking: L100 > L100-55 > HPMCAS-MG > HPMCAS-LG; selected L100 ASD gave 245% relative BA vs coarse drug and delayed Tmax (about 2.8 to 5.3 h)	Rat PK for selected L100 ASD	Strong preclinical support, but not head-to-head PK of every polymer
Nevirapine (Monschke and Wagner, 2019).	HPMCAS, HPMCP HP-50/55, Eudragit L100-55	HME ASDs evaluated in two nonsink pH-shift protocols representing standard and elevated gastric pH	Enteric-polymer ASDs remained comparatively robust to gastric-pH variation and outperformed physical mixtures/neat drug in vitro	In vitro only	Supports gastric-pH robustness; no direct PK conclusion
Celecoxib (Pöstges et al., 2022).	Eudragit L100-55, HPC-SSL, and combinations	Binary/ternary ASDs; supersaturation testing in pH 6.8 phosphate buffer over 180 min; VCM and HME processing compared	Eudragit L100-55 alone gave only transient benefit, whereas Eudragit/HPC combinations markedly prolonged supersaturation; HME ternary systems showed the strongest performance	In vitro only	Alternative or hybrid polymer strategies can outperform a single enteric polymer
ARCC-4 (Pöstges et al., 2023).	HPMCAS-L vs Eudragit L100-55	Mini-scale ASD screening and non-sink dissolution	Both polymers maintained pronounced supersaturation; no precipitation over test period	In vitro only	Useful screening evidence; no absorption claim
Efonidipine HCl (Huang et al., 2026).	HPMCAS-LG, HPC-SL, HPMCP-55, PVP K30, HPMC, Eudragit L100-55	Polymer screen in pH 6.8 buffer; optimized Eudragit L100-55 ASD evaluated by everted gut sac and beagle-dog PK	Eudragit L100-55 was the strongest polymer; optimized ASD increased apparent permeability nearly 3-fold vs crude drug and produced about 1.5-fold higher relative BA than Landel in dogs, with slower/sustained absorption	Ex vivo permeability + beagle-dog pharmacokinetics; no human PK	Recent preclinical support for a methacrylate polymer; translation to human exposure remains unproven

For candesartan cilexetil, HPMCAS-M showed the strongest overall precipitation-inhibiting performance among the polymers evaluated, although the relative effectiveness of the carriers varied with medium composition (Pinto et al., 2018). This finding demonstrates a drug- and medium-specific advantage of HPMCAS-M under the conditions examined, rather than general superiority over HPMC or Soluplus®.

In danazol ASDs, the magnitude and duration of supersaturation depended on both polymer type and drug loading. Liquid–liquid phase separation occurred before crystallization in several formulations, indicating that initial dissolved-drug concentration alone may not adequately describe formulation performance (Jackson et al., 2016).

A separate study using griseofulvin and danazol showed that inhibition of nucleation and crystal growth followed the order HPMC > PVP > Eudragit L100 under the conditions examined, although the magnitude of the effect differed between the two drugs (Ozaki et al., 2013). This result suggests that enteric functionality does not necessarily confer stronger direct precipitation inhibition and that conventional hydrophilic polymers may perform better for selected drug systems.

Differences between enteric and conventional polymers may also extend beyond precipitation inhibition. In a direct comparison of HPMCAS- and PVPVA-based ASDs of a poorly soluble model compound, both formulations showed similar apparent in vitro drug release, but the HPMCAS formulation produced better in vivo performance in dogs (Qian et al., 2012). This study illustrates that residual-matrix behavior and polymer dissolution kinetics can influence in vivo exposure even when conventional dissolution profiles appear comparable. However, the finding should be interpreted as formulation-specific evidence rather than a general advantage of HPMCAS over PVPVA.

Polymer-dependent phase behavior may also influence absorption in ways that are not predicted by conventional dissolution measurements. In naftopidil ASDs, the Eudragit L100-55 formulation showed poorer conventional dissolution performance but produced the greatest oral absorption in rats (Fukiage et al., 2022). This finding indicates that apparent dissolved concentration, phase behavior, precipitation, and absorption should be interpreted together.

Based on the evidence, direct-comparison studies do not support a simple performance hierarchy between enteric and conventional ASD polymers. Instead, several recurring patterns emerge. Enteric functionality appears most useful when premature gastric release or a gastric-to-intestinal pH transition represents a dominant formulation risk, as illustrated by studies in which delayed release preserved intestinal supersaturation. However, enteric functionality does not inherently confer stronger inhibition of nucleation or crystal growth; conventional hydrophilic polymers can perform better for selected drug systems. The comparative studies also show that conventional dissolution or apparent supersaturation rankings may not reliably predict absorption-related performance. For example, differences in residual-matrix behavior, molecularly dissolved drug, LLPS, colloidal-phase characteristics, and drug-exchange kinetics can produce membrane-flux or pharmacokinetic outcomes that differ from the ranking suggested by conventional dissolution measurements. Finally, most direct comparisons remain limited to in vitro or preclinical studies, and relatively few systems provide matched human pharmacokinetic evidence. The available literature therefore supports mechanism- and drug-specific selection of enteric polymers rather than class-based superiority.

## 4 Commercial translation and industrial adoption of enteric polymers in ASDs

The potential role of enteric polymers in modulating oral drug delivery should also be considered in the context of their use in marketed pharmaceutical products. Importantly, enteric polymers are not confined to exploratory research. They have been successfully implemented as matrix forming components in USFDA/EMA approved ASD based products designed to overcome dissolution rate/solubility limited oral absorption (Iyer et al., 2021; Newman et al., 2012; Vo et al., 2013).

This transition from experimental systems to marketed products underscores the practical relevance of the mechanistic framework discussed in earlier sections. However, the clinical performance of these products cannot be attributed to the enteric polymer alone, as commercial formulations may differ in dose, particle structure, excipient composition, manufacturing process, and drug-release behavior, while dosing conditions may also vary. A specific role for the polymer in improving dissolution, membrane transport, or pharmacokinetic exposure should be claimed only when the effect has been examined directly in supporting studies. This section discusses how enteric polymers have evolved from traditional coating excipients to supersaturation modulating excipients in selected clinically used products, and what patterns emerge from their commercial adoption. Industrial and translational studies further demonstrate the role of enteric polymers in enabling robust in vivo performance, particularly for poorly soluble and weakly basic drugs, where control of dissolution location and maintenance of supersaturation are critical determinants of systemic exposure (Kawakami, 2025; Vasconcelos et al., 2016; Vo et al., 2013).

### 4.1 USFDA/EMA approved ASD products incorporating enteric polymers

A limited but informative set of USFDA/EMA-approved oral drug products incorporating enteric polymers as matrix-forming components within amorphous or noncrystalline solid dispersions is summarized in Table 3.

**Table 3:** Selected USFDA/EMA-approved oral drug products incorporating enteric polymers in amorphous or non-crystalline formulations.^a^

Product	API	Biopharmaceutical challenge	Enteric polymer	Manufacturing approach
Zelboraf® Tablets (USFDA, CDER (2011), Application no: 202429Orig1s000, clinical pharmacology and biopharmaceutics review(s), Zelboraf tablet; Corrie et al., 2023)	Vemurafenib	Extremely low aqueous solubility; dissolution limited absorption	HPMCAS	Solvent controlled precipitation [micro precipitated bulk powder technology (MBP)]
Noxafil® DR Tablet (EMA, CHMP Noxafil, I (2014), Assessment report, Noxafil DR tablet; Corrie et al., 2023; Moseson et al., 2024)	Posaconazole	High lipophilicity; erratic exposure from oral suspension	HPMCAS	Hot melt extrusion (HME)
Kalydeco® Tablets (EMA,CHMP Kalydeco, I, 2026, Summary of product characteristics, Kalydeco film coated tablets; Corrie et al., 2023)	Ivacaftor	Poor solubility; absorption variability	HPMCAS	Spray-dried dispersion
Orkambi® Tablets (CHMP Orkambi, I. and ivacaftor, 2015, Assessment report, Orkambi film coated tablets; Corrie et al., 2023; Moseson et al., 2024)	Lumacaftor & Ivacaftor	Low aqueous solubility of both APIs (solubility-limited oral bioavailability)	HPMCAS	Spray-dried dispersion of ivacaftor; blended intragranularly with lumacaftor subsequent tablet manufacture by twin-screw wet granulation/compression
Tolsura® Capsules (FDA (US), 2018b, CDER, Application no:208901Orig1s000 multi-discipline review, Tolsura oral capsule; Ueda et al., 2026)	Itraconazole	Extremely poor solubility; variable absorption	HPMCP	Polymer-matrix formulation; manufacturing process not specified in the regulatory document reviewed; a spray-dried ASD is reported in Ueda et al. (2026).
Erleada® Tablets (FDA (US), 2018a; Corrie et al., 2023).	Apalutamide	Practically insoluble; dissolution-limited absorption	HPMCAS	Manufacturing process not specified in the regulatory/product information reviewed; spray-dried apalutamide–HPMCAS solid dispersions are described in supporting literature
Symdeko® / Symkevi® Tablets (EMA, CHMP Symkevi, I and CHMP, 2018, Assessment report, Symkevi tablets; Corrie et al., 2023; Moseson et al., 2024)	Tezacaftor/ivacaftor (Fixed dose combination) and ivacaftor tablet (co-package)	Low aqueous solubility; absorption-limited exposure	HPMCAS (Ivacaftor), and HPMC (Tezacaftor)	Spray dried ASD
Pifeltro™ Tablets (EMA, CHMP PIFELTRO, I (2018), Assessment report, Pifeltro tablets; Sauer et al., 2021)	Doravirine	Low aqueous solubility	HPMCAS	Spray drying for Doravirine HPMCAS granules
Delstrigo® Film coated bi-layer tablets (CHMP INN: doravirine / lamivudine / tenofovir and Disoproxil, 2018; Corrie et al., 2023)	Doravirine/lamivudine/ tenofovir disoproxil fumarate	Doravirine exhibits poor aqueous solubility	HPMCAS for Doravirine	Spray drying for Doravirine HPMCAS granules
Trikafta® Tablets (FDA (US), 2019, Application No. 212273Orig1s000, Multi-Discipline Review; Corrie et al., 2023; Moseson et al., 2024; Nihalani, 2025)	Elexacaftor/ivacaftor/ tezacaftor	Poor solubility of all three APIs	HPMCAS (Ivacaftor) and HPMC (Tezacaftor); elexacaftor not identified as part of the HPMCAS ASD	Spray-dried ASD
Xtandi® Tablets (FDA (US), 2020; CHMP, 2017; Nihalani, 2025; Ueda et al., 2026)	Enzalutamide	Poor aqueous solubility; dissolution-limited absorption	HPMCAS	Spray-dried ASD

a Inclusion of a product in this table indicates that an enteric polymer has been reported as part of the formulation and, where supported, that the product incorporates an amorphous/non-crystalline dispersion. Market approval does not by itself establish a specific polymer-mediated mechanism or demonstrate that the polymer alone was responsible for observed dissolution or pharmacokinetic outcomes. For combination products, polymer assignments are specified only for individual APIs where supported by the available formulation evidence.

#### 4.1.1 Translational significance: from mechanism to commercial reality

The value of enteric polymers extends well beyond mechanistic studies and is evident in several commercially available oral ASD formulations. Noxafil® (posaconazole) and Tolsura® (itraconazole) are notable examples where the combination of ASD technology and pH-responsive polymers has translated into favorable oral pharmacokinetic profiles, reduced pharmacokinetic variability, and established clinical use. However, the available clinical or pharmacokinetic findings should be interpreted in relation to the specific comparator, dose, dosage form, administration conditions, and overall formulation composition.

**Noxafil® delayed-release tablets** are an example of the clinical translation of an HPMCAS-based ASD. The oral suspension of posaconazole suffers from poor and variable absorption because of its low aqueous solubility and dependence on food intake, gastric acidity, and gastrointestinal motility as reported in the regulatory clinical pharmacology and biopharmaceutics review of Noxafil® suspension and Noxafil® DR tablets (Noxafil® DR Tablets, CDER (2013), Application no:205053Orig1s000, clinical pharmacology and biopharmaceutics review(*s*); Noxafil® Oral suspension, CDER (2005), Application no:22-003, clinical pharmacology and biopharmaceutics review(*s*)). By incorporating posaconazole into an HPMCAS-based ASD, the formulation limits drug release in the stomach and promotes dissolution in the intestinal environment. As a result, the delayed-release tablet provides higher systemic exposure than the oral suspension under fed and fasted conditions and is less affected by concomitant proton pump inhibitor (PPI) therapy than oral suspension. Noxafil, therefore demonstrates the successful clinical use of an HPMCAS-based delayed-release ASD, although its pharmacokinetic performance reflects the complete formulation and should not be attributed to the polymer alone (Chemistry review Noxafil™, Drugs FDA, 2013; DailyMed - NOXAFIL- posaconazole suspension NOXAFIL- posaconazole tablet, 2026).

**Tolsura®** is another clinically relevant example of an itraconazole formulation that highlights the advantages of combining an ASD with a pH-dependent polymer. Conventional itraconazole formulations exhibit poor dissolution and variable absorption associated with food intake and gastric acidity. In contrast, the HPMCP-based ASD maintains itraconazole in an amorphous form, limits drug release in the stomach, and promotes dissolution in the intestine. These effects may contribute to improved systemic exposure with reduced dependence on gastric acidity, illustrating how enteric polymers contribute to oral drug delivery beyond their traditional role as gastroprotective carriers (FDA (US), 2018b, CDER, Application no*:208901Orig1s000 multi-discipline review,* Tolsura oral capsule). Tolsura® and Sporanox® therefore provide a useful formulation-level comparison of two itraconazole products that differ in polymer composition, dose, dosage-form architecture, and formulation design. Pharmacokinetics of itraconazole following administration of Tolsura® and Sporanox® capsules given twice daily for 14.5 days under fed conditions in 16 healthy subjects are presented in Table 4. It is to be noted that Tolsura® contains itraconazole dispersed in an HPMCP-based polymer matrix, whereas Sporanox® contains itraconazole in amorphous drug-layered beads prepared using HPMC (Moseson et al., 2024). Despite this difference, relative bioavailability of Tolsura® 130 mg twice daily (2 × 65 mg capsules) is comparable to Sporanox® 200 mg twice daily (2 × 100 mg capsules) (FDA (US), 2018b, CDER, Application no:208901Orig1s000 multi-discipline review, Tolsura oral capsule; *TOLSURA- Itraconazole Capsule, Gelatin Coated Mayne Pharma Commercial LLC.* *TOLSURA® (Itraconazole Capsules), for Oral Use Initial U.S. Approval: 1992, 2024*). This comparison illustrates that different itraconazole formulations can produce comparable systemic exposure despite differences in dose, polymer composition, dosage form, and formulation design. The findings provide product-specific pharmacokinetic information but do not establish a general advantage of HPMCP over conventional hydrophilic polymers.

**Table 4:** Pharmacokinetics of Itraconazole Following Administration of Tolsura® and Sporanox® (Itraconazole) capsules given twice daily for 14.5 days under fed conditions in 16 healthy subjects.

Parameter^a^, ^b^	Tolsura® 130 mg twice daily (2 × 65 mg capsules)	Sporanox® 200 mg twice daily (2 × 100 mg capsules)
AUC_0-tau_ (hr*μg/ml)	15.6 ± 3.7	14.9 ± 3.8
C_trough_ (μg/ml)	1.2 ± 0.4	1.0 ± 0.3
C_max,ss_ (μg/ml)	1.6 ± 0.4	1.5 ± 0.4
T_max,ss_ (h)	7.0 (1−10)	5.0 (1–8)

a Geometric means ± standard deviation.

b T_max_ presented as median (range).

**Xtandi®** (Enzalutamide) tablets provide an example wherein the role of enteric polymer has been demonstrated. Initially, the enzalutamide was developed as a 40 mg liquid filled soft gelatin capsules (solution in caprylocaproyl polyoxylglycerides, a non-ionic hydrophilic emulsifier, and bioavailability enhancers for poorly water soluble drugs), wherein the patient has to take four capsules together to deliver 160 mg dose. Later on ASD using HPMCAS was employed and formulated as a single tablet containing 160 mg enzalutamide. Pivotal clinical study demonstrated comparative extent of absorption (AUC) between enzalutamide capsules (Xtandi® capsules) versus enzalutamide tablets (Xtandi® tablets) under fasted and fed conditions. These findings demonstrate that an HPMCAS-based ASD can achieve systemic exposure comparable to that of a lipid-based formulation while enabling the development of a convenient, single-unit tablet. This example highlights the potential of enteric polymers to support effective oral delivery of poorly water-soluble drugs through amorphous dispersion-based approaches, while also offering formulation and dosage-form advantages (FDA (US), 2020; CHMP, 2017).

### 4.2 Observed patterns in commercial adoption

Rather than treating these products as isolated examples, it is useful to examine recurring formulation and biopharmaceutical trends across approved ASD-based drug products containing enteric polymer as matrix formers.

Although multiple enteric polymers are available for pharmaceutical applications, HPMCAS has emerged as an important and widely used enteric matrix former in marketed ASD systems. This level of commercial adoption appears to arise from a combination of physicochemical tunability, mechanistic compatibility with supersaturation control, manufacturing feasibility, and accumulated translational precedent (Bapat et al., 2024; Butreddy, 2022; Moseson et al., 2024).

#### 4.2.1 Frequent application to weakly basic or highly lipophilic APIs

A notable trend among HPMCAS based ASD products is their application to weakly basic or highly lipophilic drug molecules, which are inherently prone to pH-dependent solubility limitations and precipitation as discussed in previous sections. In this context, use of enteric polymers provides a targeted strategy to modulate drug release and the availability of free drug in the region of absorption i.e., small intestine. Enteric polymers can mitigate formulation-related challenges by delaying drug release until higher pH conditions are reached, thereby supporting a more favorable dissolution–absorption balance (Nguyen et al., 2023b; Shah and Taylor, 2025).

#### 4.2.2 Grade flexibility and dissolution modulation

HPMCAS is commercially available in multiple substitution grades (commonly designated L, M, and H), which differ in substitution pattern and exhibit grade dependent dissolution behavior, including progressively higher dissolution pH thresholds with increasing hydrophobic substitution. This enables formulation specific tuning of release onset and supersaturation generation. In practice, such grade dependent behavior provides a practical formulation lever for aligning drug release with gastrointestinal site of drug delivery (Shah and Taylor, 2025; Tanno et al., 2004).

#### 4.2.3 Amphiphilic substitution pattern and supersaturation stabilization

The coexistence of hydrophobic acetyl groups and ionizable succinoyl moieties confers amphiphilic character and pH-dependent solubility, supporting drug–polymer interactions and interfacial stabilization of drug-rich phases under appropriate intestinal conditions. Mechanistic studies demonstrate that HPMCAS substitution chemistry and grade influence crystallization inhibition, drug-rich phase formation, and dissolution dynamics (Bapat et al., 2024; Shah and Taylor, 2025; Ueda and Taylor, 2020). This behavior reflects the combined influence of polymer chemistry and environmental pH, rather than a single dominant mechanism.

#### 4.2.4 Manufacturing compatibility and platform maturity

Successful translation of ASD formulations requires not only drug–polymer compatibility but also compatibility with scalable manufacturing processes. In some cases, the polymer must also withstand the thermal and mechanical stresses associated with manufacturing, particularly during hot-melt extrusion. HPMCAS is highly compatible with spray drying and, in selected systems, hot melt extrusion, supporting scalable production of homogeneous dispersions with reproducible performance. From an industrial perspective, this compatibility is a key factor enabling consistent large-scale manufacturing. Multiple USFDA/EMA approved products document HPMCAS as a dispersion matrix former, reinforcing regulatory familiarity and translational experience for subsequent development programs (Bhujbal et al., 2021; Iyer et al., 2021; Moseson et al., 2024).

### 4.3 Understanding the limited commercial translation of HPMCP-based ASDs

HPMCP shares pH-dependent solubility characteristics with HPMCAS and has long been used as an enteric coating polymer. However, relative to HPMCAS, it appears less frequently as a primary matrix former in USFDA/EMA approved ASD products, reflecting differences in historical platform development and accumulated translational precedent rather than an intrinsic mechanistic limitation (Butreddy, 2022; Moseson et al., 2024).

One contributing factor may be the greater depth of mechanistic and translational literature centered on HPMCAS based ASDs, including grade dependent dissolution control and extensive evaluation in supersaturation and colloid-mediated dissolution paradigms. In addition, commercial programmes and process development toolkits for spray dried dispersions have been heavily optimized around HPMCAS across multiple approved products, which can bias industrial selection toward the polymer with greater precedent and platform maturity (Bapat et al., 2024; Iyer et al., 2021; Moseson et al., 2024). From an industrial standpoint, this creates a self-reinforcing cycle in which prior success drives continued selection.

Importantly, the lower representation of HPMCP in marketed ASD based drug products should not be interpreted as inferiority. Both polymers are pH-responsive and capable of modulating supersaturation behavior, but commercial adoption patterns often reflect path dependence, driven by platform familiarity, manufacturing precedent, and regulatory experience rather than purely mechanistic superiority (Butreddy, 2022; Iyer et al., 2021). Accordingly, polymer selection in ASD development should be guided by system-specific considerations rather than historical usage patterns alone.

### 4.4 Limitations and practical considerations

Enteric polymer-based ASDs are subject to important formulation and evaluation related limitations (Gan et al., 2023; Kostewicz et al., 2014). Drug–polymer compatibility and system-specific interactions remain critical determinants of performance, as supersaturation stabilization is highly dependent on molecular complementarity rather than polymer class alone (Ilevbare et al., 2012; Warren et al., 2010). This underscores that formulation performance cannot be generalized across drugs and must be evaluated on a case-by-case basis. Published studies have also demonstrated that drug–polymer compatibility cannot be assumed even among enteric polymers; for example, atorvastatin was found to be incompatible with HPMCP (Mittal et al., 2025).

Furthermore, evaluation of enteric polymer-based ASDs presents additional challenges. Predicting in vivo performance based solely on the in vitro data remains challenging without appropriate biorelevant methodologies. These considerations highlight the need for integrated evaluation approaches combining biorelevant dissolution testing, flux based assessments, and stability studies to ensure robust and reproducible in vivo performance (Iyer et al., 2021; Mudie et al., 2010).

Application of conventional two stage acid resistance dissolution testing to enteric polymer-based ASDs intended to enhance bioavailability may introduce additional analytical and operational burden during quality control evaluation (USFDA, 2018, Dissolution Methods Database; General Chapter 711 Dissolution | USP-NF, 2012). Unlike classical enteric coated systems designed to protect acid labile drugs from gastric degradation, many ASD formulations employ enteric polymers primarily to spatially modulate supersaturation and precipitation behavior rather than to completely prevent gastric exposure. In such systems, strict binary assessment of acid stage resistance may not fully capture the intended in vivo function of the formulation (Jørgensen et al., 2023). This highlights the need for more mechanistically relevant dissolution methodologies capable of evaluating both release behavior and supersaturation dynamics under biorelevant conditions.

## 5 Manufacturing strategies for enteric polymer-based ASDs

The successful translation of enteric polymer-based ASDs into clinically viable products is closely linked to the selection of appropriate manufacturing platforms, which must be compatible with both the physicochemical properties of the drug and the functional characteristics of the polymer. In particular, the pH-dependent solubility, amphiphilic structure, and thermal properties of enteric polymers impose distinct constraints and opportunities that ultimately influence the physicochemical attributes, stability, and in vivo performance of the resulting ASD (Corrie et al., 2023; Vehring, 2007). In practice, these considerations require careful alignment between formulation objectives and process capabilities.

### 5.1 Spray drying as the preferred platform

Spray drying is a widely used manufacturing approach for enteric polymer-based ASDs. The technique enables the formation of homogeneous molecular dispersions through rapid solvent evaporation, effectively trapping the drug in an amorphous state and minimizing phase separation (Li and Taylor, 2018; Vehring, 2007). Spray drying is compatible with enteric polymers due to their solubility in organic solvent systems and their ability to form stable dispersions during rapid solidification. The resulting particles may exhibit favorable particle size and surface area, morphology, and dissolution characteristics, supporting robust supersaturation generation and maintenance (Corrie et al., 2023; Li and Taylor, 2018). However, these attributes are formulation- and process-dependent and should not be assumed for all enteric polymer-based ASDs. Spray drying is widely regarded as the most versatile and scalable platform for enteric polymer-based ASD development.

### 5.2 Hot-melt extrusion (HME): opportunities and limitations

HME represents an alternative solvent free (green) manufacturing platform that has been applied to selected enteric polymer-based systems. However, its applicability is often constrained by the thermal properties of both the drug as well as the polymer, including their Tg and degradation thresholds (Repka et al., 2018). While HPMCAS can be processed via HME under optimized conditions, its relatively high Tg and potential susceptibility to thermal and hydrolytic degradation may necessitate the use of plasticizers or modified processing conditions. Consequently, HME generally requires more careful optimization of processing temperature, residence time, and polymer grade selection than spray drying (Maniruzzaman et al., 2012; Repka et al., 2018). The thermal and shear history experienced during HME may influence molecular mobility, polymer relaxation, and the long-term physical stability of the ASD. Accordingly, the choice between spray drying and HME is often driven by the balance between drug-polymer stability, process feasibility and desired microstructural attributes, as illustrated in Fig. 3. .

**Fig. 3:**
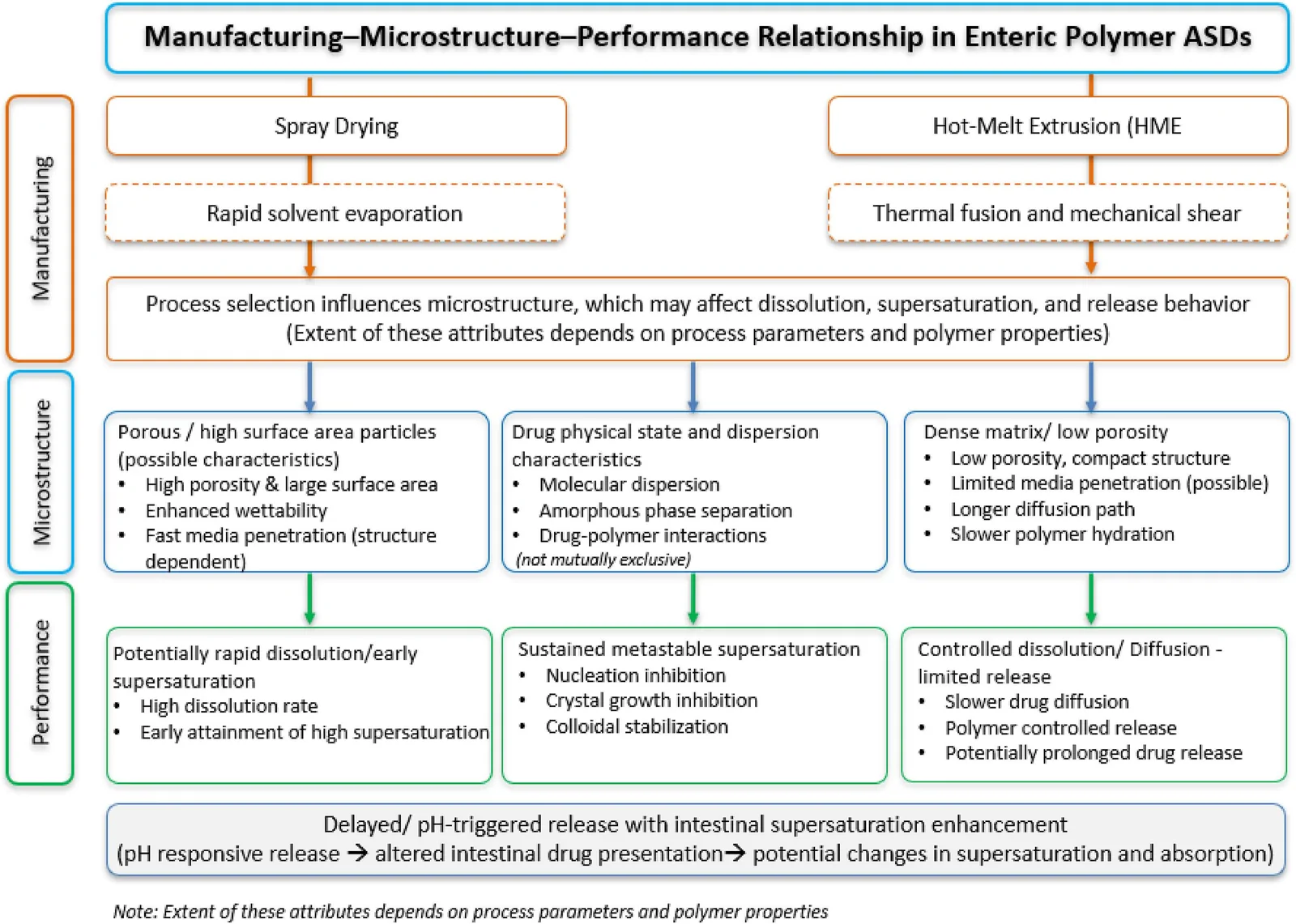
Conceptual relationship between manufacturing processes, ASD microstructure, and potential biopharmaceutical performance of enteric polymer-based ASDs. Spray drying and hot-melt extrusion can generate distinct particle and matrix characteristics, which may influence drug–polymer interactions, dissolution, supersaturation, and drug-release behavior. The extent and direction of these effects depend on the drug–polymer combination, material attributes, process parameters, and downstream processing conditions. The proposed relationships are conceptual and require experimental validation for individual formulations. Original conceptual illustration prepared by the authors specifically for this review; not reproduced or adapted from previously published material.

Emerging technologies, including KinetiSol processing, electrospinning, and continuous manufacturing approaches are also being explored for enteric polymer-based ASDs. Although industrial experience with these platforms remains limited, these may expand the processing window for thermally sensitive formulations and provide greater manufacturing flexibility. However, their applicability to specific enteric polymers and their ability to improve product performance require further systematic evaluation.

### 5.3 Solvent selection and polymer–process compatibility

The selection of solvent systems in spray drying is a critical determinant of ASD quality, particularly for enteric polymers. HPMCAS requires appropriate organic solvents or solvent blends to achieve adequate dissolution and uniform drug–polymer mixing prior to atomization (Bhugra and Pikal, 2008; Vehring, 2007). Solvent selection can influence particle formation kinetics, drug–polymer mixing, residual solvent content, and ultimately the homogeneity and physical stability of the resulting dispersion. Inadequate solvent selection may lead to phase separation, incomplete amorphization, or undesirable particle characteristics, potentially compromising supersaturation performance. Therefore, solvent–polymer compatibility must be carefully evaluated during early process development (Bhugra and Pikal, 2008; Leuner and Dressman, 2000).

### 5.4 Stability considerations during processing

Enteric polymers introduce unique stability considerations during manufacturing. These include:

• Thermal degradation risks, particularly under HME conditions (Repka et al., 2018).
• Potential hydrolysis of ester functional groups (e.g., succinoyl or phthalyl moieties) under high humidity or temperature (Bhugra and Pikal, 2008).
• Moisture-induced plasticization, which can reduce Tg and promote phase separation (Chen et al., 2018; Krummnow et al., 2023; Lehmkemper et al., 2017; Patel and Serajuddin, 2022).

Taken together, these factors necessitate careful control of processing temperature, humidity, and residence time, particularly during HME, as well as appropriate selection of packaging and storage conditions. These stability considerations further reinforce the importance of aligning polymer chemistry with manufacturing strategy in ASD development (Chen et al., 2018; Krummnow et al., 2023; Lehmkemper et al., 2017; Patel and Serajuddin, 2022; Van Den Mooter, 2012). The major manufacturing- and material-related risk factors, their potential biopharmaceutical consequences, and the corresponding development controls are summarized in Table 5.

**Table 5:** Manufacturing and material-related risk factors in enteric polymer-based ASDs and recommended development controls.

Process / variable	Material or microstructural effect	Potential biopharmaceutical consequence	Recommended development control
Spray drying: solvent system / feed solids	May influence drug–polymer mixing, viscosity, droplet formation, residual solvent, and particle formation	Potential phase separation, variable amorphous content, altered hydration, and changes in dissolution	Verify complete feed solubilization; map residual solvent and solid-state homogeneity (Bhujbal et al., 2021; Paudel et al., 2013; Vehring, 2007).
Spray drying: atomization and drying rate	Changes particle size, porosity, surface composition and density	Altered particle hydration, pH-shift release, and supersaturation behavior	Scale-up drying history and particle attributes, not only nominal temperature and feed rate
HME: temperature / shear / residence time	Changes melt viscosity, degradation risk, polymer ester integrity and dispersion state	Potential changes in enteric dissolution behavior, chemical stability, and drug-release characteristics	Establish thermal/shear design space; monitor free acids, impurities, solid-state (Repka et al., 2018; Sarode et al., 2014).
HME: plasticizer or surfactant	Lowers processing temperature but changes miscibility and phase behavior	May alter drug activity, phase behavior, drug–polymer interactions, or precipitation inhibition	Evaluate the effect of the additive on pH-shift dissolution, molecularly dissolved drug, precipitation kinetics, and membrane flux, not only torque or processability.
Downstream milling / compression	Changes surface area, densification, wettability and tablet disintegration	May alter surface area, wetting, disintegration, and the accessibility of the polymer-controlled release mechanism	Assess intermediate and final dosage forms using the same biorelevant transfer and pH-shift dissolution protocols
Moisture exposure / storage	Plasticization and possible phase separation, crystallization, or polymer hydrolysis under relevant conditions	Loss of physical stability or changed release profile	Controlling relative humidity during processing and storage, selecting less hygroscopic polymers, optimizing drug–polymer composition, and using moisture-protective packaging to limit water uptake and preserve physical stability. (Chen et al., 2018; Lehmkemper et al., 2017; Patel et al., 2022; Patel and Serajuddin, 2022).

### 5.5 Process–performance interrelationship

Manufacturing conditions influence particle morphology, drug–polymer dispersion, matrix structure, and, consequently, the dissolution and supersaturation behavior of enteric-polymer ASDs (Corrie et al., 2023; Li and Taylor, 2018; Van Den Mooter, 2012). These effects depend on the drug–polymer combination, material attributes, process parameters, and downstream processing conditions (Bhugra and Pikal, 2008; Leuner and Dressman, 2000). Therefore, process selection should be integrated into formulation design rather than treated as an independent engineering decision. The resulting process–structure–performance relationship is illustrated in Fig. 3 and requires experimental validation for individual formulations.

## 6 Mechanism-based framework for evaluating the suitability of enteric polymers in ASD systems

The selection of enteric polymers for ASD systems should be guided by the drug's physicochemical and biopharmaceutical properties, gastrointestinal physiology, and formulation objectives rather than by empirical polymer screening alone (Taylor and Zhang, 2016). Their suitability should therefore be evaluated as part of a risk-informed, mechanism-based formulation strategy, as illustrated in Fig. 4.

**Fig. 4:**
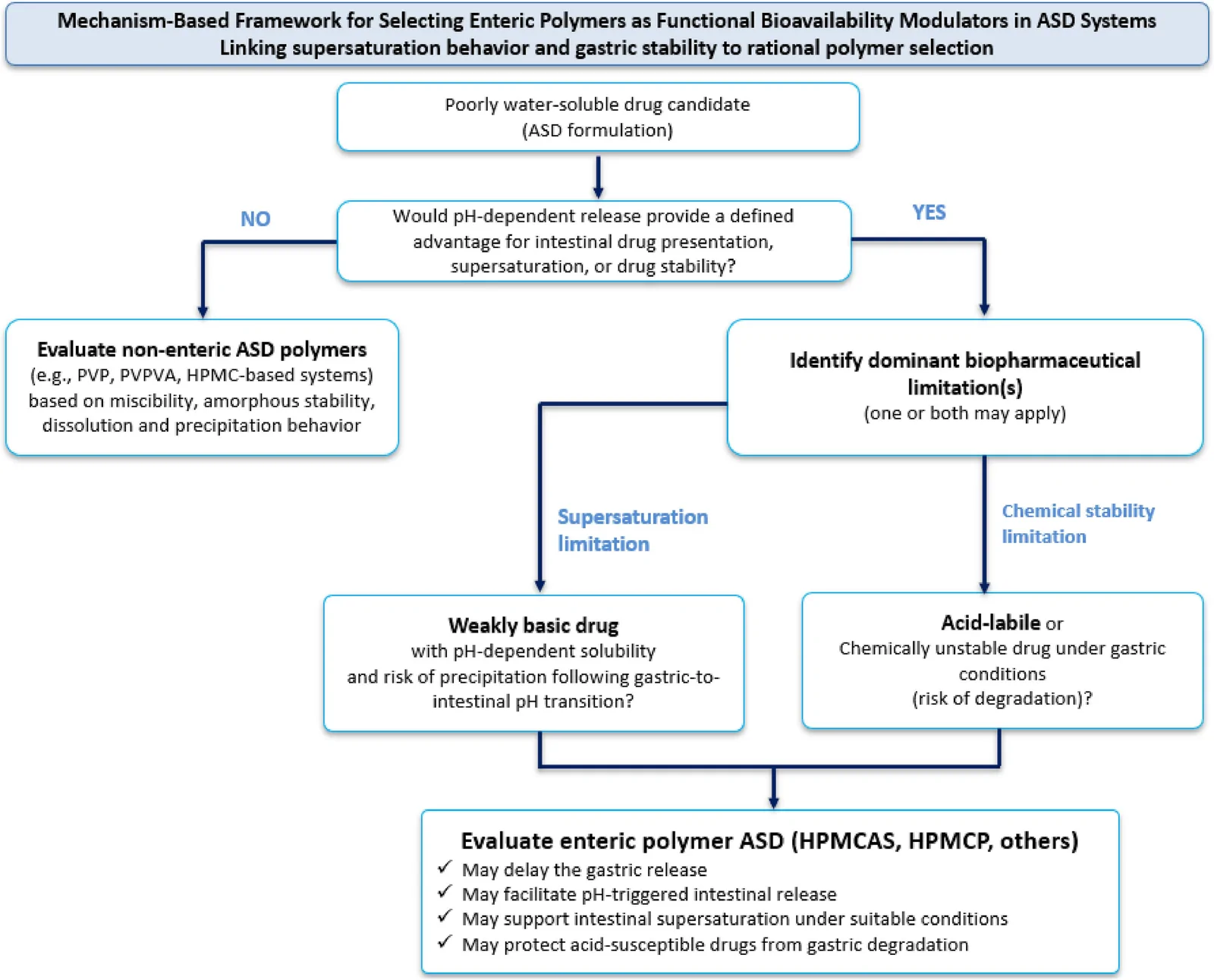
Mechanistic decision pathway for selecting enteric polymers in ASD systems. Original conceptual illustration prepared by the authors specifically for this review; not reproduced or adapted from previously published material.

To translate these mechanistic considerations into a practical formulation-development strategy, Table 6 presents a decision framework for assessing whether an enteric polymer is likely to provide a meaningful advantage over a conventional ASD polymer. The framework considers drug-specific physicochemical and biopharmaceutical properties, polymer dissolution behavior, supersaturation and precipitation kinetics, permeability, physiological variability, drug loading, and manufacturing feasibility. Each decision point is linked to an experimental approach and potential alternative strategy if the expected benefit of an enteric polymer is not demonstrated. This framework is intended to guide hypothesis-driven formulation development rather than serve as a validated predictive algorithm.

**Table 6:** Decision framework for evaluating the suitability of enteric polymers in amorphous solid dispersions.

Decision question	Evidence favoring an enteric ASD	Risk / failure signal	Preferred experiment	Alternative if unfavorable
1. Does the drug exhibit pH-dependent solubility, ionization, or pKa-related precipitation risk?	A pH-dependent solubility profile, substantial changes in drug ionization across gastrointestinal pH conditions, or precipitation risk associated with gastric-to-intestinal pH transition.	Minimal pH-dependent solubility change or no meaningful precipitation risk across physiologically relevant gastrointestinal pH conditions.	Determine the drug's pKa and pH–solubility profile, followed by biorelevant pH-shift dissolution and precipitation studies.	Consider a non-enteric ASD or another formulation strategy if pH-dependent release does not provide a demonstrable biopharmaceutical advantage.
2. Does gastric-to-intestinal pH change create a solubility/precipitation problem?	Pronounced pH-dependent solubility; rapid precipitation after pH shift; weak-base salt/free-base conversion associated with precipitation risk	Little pH-dependence or precipitation that is slow relative to absorption process	Intrinsic and amorphous solubility as a function of pH; pH-shift induction time and precipitation profile	Conventional hydrophilic ASD if rapid release is desirable
3. Is the drug chemically unstable in gastric conditions?	Meaningful acid-catalyzed degradation during physiologically relevant gastric residence	Adequate gastric stability	Time-dependent degradation in simulated gastric media across realistic pH	Non-enteric ASD if gastric protection is unnecessary; conventional enteric coating if gastric protection, rather than supersaturation control, is the primary objective
4. Can the selected polymer dissolve within the absorption window?	Polymer dissolution and drug release occur at the intended proximal intestinal pH and buffer capacity	High-threshold grade, low buffer capacity, or narrow proximal intestinal window resulting in delayed or incomplete release	Dynamic pH/buffer with measurement of polymer dissolution and drug release from the dosage form	Lower-threshold grade/polymer; pH-independent ASD; other enabling technology
5. Can the drug-polymer pair maintain a useful state after release?	Delayed nucleation/crystal growth; adequate molecularly dissolved drug concentration; sustained or increased membrane flux under relevant conditions	Excessive drug–polymer association that reduces the molecularly dissolved fraction, or failure of the polymer to inhibit precipitation	Precipitation kinetics, amorphous solubility/activity, molecularly dissolved drug, and flux/permeation	Different polymer/grade or ternary polymer combination
6. Is crystallization tendency compatible with practical drug loading?	No unacceptable phase separation or crystallization at the target drug loading; acceptable storage stability	Phase separation or crystallization at the required drug loading	DSC/PXRD/spectroscopy; accelerated humidity- and temperature-based stability studies	Lower loading, ternary ASD, co-amorphous system, nanocrystal or lipid formulation
7. Is dose compatible with the amount of polymer required?	Acceptable tablet or capsule size at the polymer-to-drug ratio required for physical stability and precipitation control	Dose burden makes the required polymer-to-drug ratio impractical	Dose/solubility assessment plus ASD loading screen	Lower drug loading, ternary ASD, lipid system, nanocrystal, salt/cocrystal, enabling combination (drug-dependent cases)
8. Is permeability sufficient to benefit from supersaturation?	Solubility-limited and sufficiently permeable drug; increased molecularly dissolved concentration is associated with increased membrane flux	Permeability-limited compound; increased apparent concentration does not increase molecularly driven membrane flux	Dissolution–permeation or membrane-flux testing, with distinction between molecularly dissolved and colloid-associated drug	Permeability-focused strategy or different route/technology
9. Is performance robust to physiological variability?	Acceptable release and supersaturation performance across relevant fasted/fed conditions, pH and buffer-capacity ranges, bile components, and acid-reducing conditions	Large release delays or incomplete release under physiologically plausible gastrointestinal conditions	Biorelevant transfer studies; sensitivity analysis and PBPK modelling where justified	Lower-threshold polymer or less pH-dependent formulation
10. Is the polymer compatible with the manufacturing process?	Stable during spray drying/HME; reproducible microstructure and downstream performance	Thermal or chemical degradation, polymer hydrolysis where relevant, residual-solvent issues, or poor powder properties	Process-stress studies, scale-up comparability, post-processing pH-shift dissolution	Switch process, polymer grade, or formulation platform

As summarized in Table 6, enteric polymers may be particularly relevant when gastric exposure is associated with chemical or physical instability, premature precipitation, loss of absorption-relevant supersaturation, or an inappropriate dissolution window before the drug reaches its principal absorption region (Kostewicz et al., 2004; Maderuelo et al., 2019; Nguyen et al., 2023b). Conversely, when gastric exposure does not adversely affect drug stability, dissolution, or supersaturation behavior, conventional ASD polymers such as PVP, PVPVA, HPMC, or PVA may provide adequate performance, provided that acceptable drug–polymer compatibility, physical stability, and manufacturing feasibility are demonstrated (Qian et al., 2010; Vora et al., 2016).

### 6.1 Key mechanistic and development considerations

A principal indication for enteric-polymer selection is pronounced pH-dependent drug solubility accompanied by a high risk of precipitation during gastric-to-intestinal transfer (Kostewicz et al., 2004; Taylor and Zhang, 2016). This behavior is particularly relevant to weakly basic drugs (General Chapter 711 Dissolution | USP-NF, 2012;USFDA, 2018, Dissolution Methods Database; Kostewicz et al., 2004). In such cases, modulating drug release until intestinal conditions may reduce premature precipitation and improve the temporal relationship between dissolution and absorption. The suitability of this strategy depends on the magnitude of the pH-related solubility change, precipitation kinetics, intestinal release rate, and location of the drug's absorption window. The decision framework in Table 6 integrates these considerations into a sequence of formulation-development questions and identifies potential alternatives when an enteric polymer does not provide a clearly demonstrated advantage.

### 6.2 Practical selection principle

Enteric polymers are most appropriately selected when delayed gastric release offers a defined mechanistic advantage through:

• Preservation of absorption-relevant intestinal supersaturation for drugs with pH-dependent solubility and a high risk of precipitation (Kostewicz et al., 2004; Taylor and Zhang, 2016).
• Protection of drugs that are chemically unstable under gastric conditions (Maderuelo et al., 2019).

When gastric exposure does not materially compromise drug stability or absorption-relevant dissolution behavior, conventional ASD polymers may provide adequate performance without the additional formulation complexity and physiological considerations associated with enteric systems (General Chapter 711 Dissolution | USP-NF, 2012; USFDA, 2018, Dissolution Methods Database; Qian et al., 2010). Enteric-polymer selection should therefore balance the expected biopharmaceutical benefit against formulation complexity and should be supported by drug-specific dissolution, stability, and, where appropriate, permeability or pharmacokinetic evidence.

## 7 Limitations and risk factors in enteric polymer-based ASDs

While enteric polymer-based ASDs may provide advantages for selected poorly soluble drugs, their application is associated with several limitations and risk factors that must be carefully considered. A comprehensive understanding of these challenges is essential for rational design and successful clinical translation. In practice, these limitations often arise from the interaction between formulation design and physiological variability.

### 7.1 Food effect and gastrointestinal variability

A key limitation of enteric polymer-based systems is their susceptibility to physiological variability within the gastrointestinal tract. Food intake can alter gastric pH, gastric emptying time, bile-salt concentration, intestinal fluid composition, and luminal buffer capacity, all of which may influence polymer dissolution, drug release, precipitation, and absorption. Prolonged gastric residence in the fed state may delay intestinal release, whereas increased bile-salt concentrations may enhance drug solubilization but also modify the formation and persistence of drug-rich phases (Butreddy, 2022).

In addition to food effects, inter- and intra-patient variations in gastrointestinal pH may alter the timing and completeness of enteric-polymer dissolution. Changes associated with age, disease, or acid-reducing medications can modify gastric-phase behavior and subsequent intestinal drug release. Relatively low intestinal pH or limited buffer capacity may delay polymer dissolution, shift drug release toward more distal intestinal regions, or reduce release within the available absorption window (Nguyen et al., 2023b; Vehring, 2007). These factors may increase variability in dissolution and systemic exposure. Accordingly, enteric-polymer ASDs should be evaluated under physiologically relevant fasted and fed conditions and across plausible gastric and intestinal pH and buffer-capacity ranges.

### 7.2 Delayed T_max_ and onset of action

Depending on their composition and intended release behavior, enteric polymer-based systems may delay drug release until the formulation reaches the intestinal environment. While this property is beneficial for protecting acid labile drugs and avoiding gastric precipitation, it may lead to delayed Tmax and slower onset of action. This limitation is particularly relevant for drugs requiring rapid therapeutic response, where physiological factors such as gastric emptying and intestinal transit play a key role in determining the rate of drug absorption (Mudie et al., 2010). Accordingly, the suitability of enteric systems must be evaluated in the context of therapeutic requirements and the intended drug release profile.

### 7.3 Risk of intestinal precipitation

Although enteric polymers may support supersaturation generation in the intestinal environment, this supersaturated state is inherently metastable. Inadequate stabilization may lead to precipitation before absorption occurs, thereby reducing the concentration of drug available for absorption. The balance between supersaturation generation and precipitation inhibition is highly sensitive to formulation composition, polymer properties, and intestinal conditions. Failure to adequately control precipitation kinetics remains a key limitation of ASD systems (Indulkar et al., 2016; Kong et al., 2023). Importantly, the presence of drug-rich colloidal or other drug-containing phases should not be interpreted as direct evidence of improved absorption. Their contribution to drug uptake depends on the extent of molecularly dissolved drug, drug release at relevant interfaces, and the relationship between apparent drug concentration and membrane flux.

### 7.4 Cost considerations compared to conventional polymers

The selection of enteric polymers must also consider practical formulation and development factors. Compared to more commonly used polymers such as PVP or PVPVA, enteric polymers may introduce additional formulation complexity related to pH-dependent solubility and processing requirements. In cases where enteric functionality does not provide a clear biopharmaceutical advantage, conventional polymers may remain preferable from a development and manufacturing perspective. Thus, cost–benefit considerations play an important role in polymer selection.

### 7.5 Scale-up and manufacturing challenges

Scaling up enteric polymer-based ASD manufacturing requires preservation of the drug–polymer microstructure and dissolution performance established at development scale. During spray drying, changes in equipment geometry, atomization, feed rate, solids concentration, gas flow, and drying kinetics can alter particle morphology, density, porosity, surface composition, residual solvent, and drug–polymer homogeneity. These changes may influence powder handling, downstream processing, dissolution, supersaturation generation, and long-term stability. Commercial spray drying also requires effective solvent recovery and control of flammability and environmental exposure when organic solvents are used (Bhugra and Pikal, 2008; Maniruzzaman et al., 2012).

During hot-melt extrusion, scale-up can alter thermal and shear history, residence-time distribution, mixing efficiency, and specific mechanical energy. For enteric polymers with relatively high glass-transition temperatures or narrow processing windows, inadequate processing may cause incomplete amorphization, whereas excessive heat, shear, or moisture may promote drug degradation, polymer hydrolysis, or changes in pH-dependent dissolution. Reproducibility of extrudate cooling, milling, flow, compression, and content uniformity must also be maintained during downstream manufacture (Bhugra and Pikal, 2008; Maniruzzaman et al., 2012).

Commercial translation therefore requires control of critical material attributes including polymer grade, viscosity, substitution pattern, and moisture content and critical process parameters that govern amorphous content, microstructure, residual solvent, dissolution, supersaturation, and stability. Process analytical technology and scale-relevant comparability studies can help ensure consistent product performance (Bhugra and Pikal, 2008; Maniruzzaman et al., 2012).

### 7.6 Hydrolysis and chemical stability risks

Moisture- and temperature-associated hydrolysis of polymer ester groups may alter polymer functionality and ASD stability under certain conditions. Accordingly, storage conditions, packaging, and environmental exposure should be controlled during product development and lifecycle management (Chen et al., 2018; Lehmkemper et al., 2017; Patel et al., 2022).

Collectively, these limitations highlight the importance of a risk-based formulation strategy, wherein the potential benefits of enteric polymer-based ASDs are carefully balanced against variability in gastrointestinal conditions, stability constraints, and manufacturing considerations. In practice, successful implementation requires alignment between drug properties, polymer functionality, and process design to ensure consistent and predictable in vivo performance.

## 8 Future perspectives and conclusions

### 8.1 Future perspectives

Enteric polymer-based ASDs have evolved from conventional enteric coating systems into multifunctional platforms capable of influencing the site and timing of drug release, stabilizing supersaturation, and improving intestinal drug presentation. Despite substantial progress, further research is required to establish when enteric polymers provide clinically meaningful improvements in oral drug exposure rather than functioning primarily as protective or delayed-release excipients. Future advances will depend on closer integration of polymer design, biorelevant performance testing, predictive modelling, manufacturing control, and clinical validation.

One important direction is the rational development of next-generation enteric polymers with tailored physicochemical properties. Current polymers such as HPMCAS and HPMCP provide established pH-responsive functionality, but their performance can be constrained by fixed dissolution-pH thresholds, limited chemical tunability, moisture sensitivity, and drug-dependent compatibility. Polymers with adjustable ionization profiles, controlled substitution patterns, improved moisture resistance, and optimized hydrophobic and hydrogen-bonding functionality could provide more precise control over polymer dissolution, drug–polymer association, precipitation inhibition, and interfacial stabilization. Establishing quantitative relationships among polymer molecular weight, substitution pattern, ionization, chain conformation, drug affinity, and in vivo performance will be important for moving polymer selection from empirical screening toward mechanism-based design.

Future translational studies should also distinguish the contribution of the enteric polymer from other formulation variables. Compositionally matched, head-to-head comparisons of enteric- and non-enteric-polymer ASDs are needed in which polymer type or grade is the principal experimental variable. Such studies could clarify the relative contributions of delayed gastric release, intestinal supersaturation generation, precipitation inhibition, drug-rich phase stabilization, particle microstructure, and amorphization to systemic exposure. Prospective evaluation against pharmacokinetic outcomes, including AUC, C_max_, T_max_, food effect, and inter-subject variability, will be necessary to establish when enteric polymers contribute to clinically meaningful pharmacokinetic advantages.

The integration of mechanistic modelling and predictive tools represents another important research priority. Physiologically based pharmacokinetic modelling, in vitro*–*in vivo correlation, and biorelevant dissolution–permeation testing may improve prediction of enteric polymer-based ASD performance under dynamic gastrointestinal conditions. In vitro studies should move beyond maximum apparent drug concentration and evaluate the onset, duration, and regional location of supersaturation, precipitation kinetics, molecularly dissolved drug, membrane flux, and cumulative permeation. These measurements should be interpreted in relation to gastrointestinal pH, buffer capacity, fluid volume, bile salts, phospholipids, gastric emptying, intestinal transit, food intake, disease state, and concomitant medications. Integrating these data into mechanistic absorption models may reduce empirical formulation screening and support selection of formulations that remain robust across physiologically relevant conditions.

Further work is needed to establish when drug-rich colloidal phases support membrane transport and when they instead retain drug in poorly permeable states. Linking colloidal characteristics with free-drug concentration, membrane flux, and pharmacokinetic exposure will strengthen the mechanistic basis for formulation design.

Advanced hybrid delivery systems may further expand the functionality of enteric polymers. Future formulations may combine pH-responsive polymers with lipid-based systems, polymeric or lipid nanoparticles, cubosomes, vesicular carriers, co-amorphous systems, or other nanostructured platforms. However, their use in oral delivery remains limited by gastrointestinal instability, loading capacity, reproducibility, scale-up, safety, and regulatory complexity. These technologies are therefore better viewed as complementary platforms rather than universal replacements for enteric polymer-based ASDs. Direct comparisons with simpler formulations will be necessary to determine whether their added complexity produces clinically meaningful benefits.

From a manufacturing perspective, continuous processing and advanced process-control strategies may improve scalability and reproducibility. Continuous spray drying, twin-screw extrusion with real-time monitoring, and process analytical technology can provide tighter control over particle morphology, drug–polymer homogeneity, residual solvent, amorphous content, and downstream processability. Future work should establish clinically relevant critical material attributes and critical process parameters that connect polymer variability and ASD microstructure with dissolution, supersaturation, permeability, stability, and in vivo performance. Such process–structure–performance relationships will be essential for robust commercial translation.

Patient-centred formulation design should also receive greater attention. Variability in gastrointestinal physiology, age, disease state, food intake, acid-reducing medications, and intestinal transit may alter the timing and extent of polymer dissolution and drug absorption. Formulations should therefore be evaluated across relevant fasted and fed conditions and under physiologically plausible pH, buffer-capacity, motility, and transit scenarios. Personalized or adaptable formulations supported by PBPK modelling and biorelevant testing may ultimately help identify polymer grades and release profiles that are better suited to specific patient populations.

Overall, future research should focus not only on demonstrating improved dissolution or prolonged apparent supersaturation, but also on establishing whether enteric polymers produce reproducible, clinically meaningful improvements in exposure, absorption consistency, food-effect robustness, and therapeutic performance.

### 8.2 Conclusions

Enteric polymers represent a versatile class of functional excipients in the design of ASDs for oral drug delivery. This review highlights the mechanistic basis by which enteric polymers may influence drug release, supersaturation, precipitation behavior and intestinal drug presentation. Comparative analysis demonstrates that, while conventional polymers remain effective in many cases, enteric polymers may provide distinct advantages under conditions involving pH-dependent solubility, gastric instability, or the need for targeted intestinal delivery. Importantly, these advantages are context-dependent and must be aligned with drug-specific and physiological factors.

The manufacturing and processing considerations associated with enteric polymer-based ASDs underscore the importance of aligning polymer chemistry with process design. Furthermore, the decision framework presented in this review offers a practical, mechanistically grounded approach for selecting enteric polymers based on drug properties and formulation objectives. This framework provides a basis for more rational and predictive formulation development. At the same time, several limitations and risk factors, including variability in gastrointestinal conditions, delayed drug release, moisture sensitivity, and scale-up challenges, highlight the need for careful formulation design and risk-based evaluation.

Overall, the performance of an enteric-polymer ASD is not determined by enteric functionality alone but emerges from the interaction among drug properties, polymer chemistry and grade, formulation composition and drug loading, processing conditions and resulting microstructure, and the gastrointestinal environment. Drug ionization, pH-dependent solubility, crystallization tendency, permeability and absorption window must therefore be considered together with polymer dissolution behavior, drug–polymer interactions, manufacturing feasibility, gastrointestinal pH, buffer capacity, gastric emptying and intestinal transit. Enteric polymers should consequently not be regarded as universally superior to conventional ASD carriers. Their use is most justified when pH-dependent release or polymer-specific stabilization addresses a defined biopharmaceutical risk and when the resulting advantage is demonstrated using formulation-relevant dissolution, phase-behavior, permeation and, where appropriate, pharmacokinetic evidence. From this perspective, enteric polymers are best viewed as one component of a broader mechanism-based formulation strategy rather than as an intrinsically superior polymer class.

## Declaration of generative AI and AI-assisted technologies in the manuscript preparation process

During the preparation of this work, the authors used ChatGPT (OpenAI) to assist with language refinement, sentence clarity, and readability in selected portions of the manuscript. Generative AI was not used to generate, reproduce, adapt, or modify any figures or the graphical abstract. The scientific content, interpretation of the literature, conclusions, and final text were reviewed and approved by the authors. The authors take full responsibility for the content of the article.

## CRediT authorship contribution statement

**Vishvesh Joshi:** Writing – original draft, Methodology, Investigation, Formal analysis, Conceptualization. **Lokesh Yadav:** Writing – original draft, Methodology, Investigation, Formal analysis, Conceptualization. **Yalamanda Varla:** Writing – review & editing, Formal analysis. **Pranav Patel:** Writing – review & editing. **Yagni Shah:** Writing – review & editing. **Arvee Prajapati:** Investigation. **Vijaykumar Chaudhary:** Investigation. **Rikin Patel:** Writing – review & editing, Formal analysis. **Praveen Thaggikuppe Krishnamurthy:** Writing – review & editing. **Sonam Patel:** Writing – review & editing, Supervision, Formal analysis. **Derajram Benival:** Writing – review & editing, Supervision, Project administration, Investigation, Formal analysis, Conceptualization.

## Declaration of competing interest

The authors declare that they have no known competing financial interests or personal relationships that could have appeared to influence the work reported in this paper.

## Data Availability

No new datasets were generated or analyzed for this narrative review.
